# Intestinal toxicity of functionalized polystyrene nanoplastics in NOD2 models of Crohn’s disease susceptibility

**DOI:** 10.3389/ftox.2026.1839932

**Published:** 2026-07-21

**Authors:** Manon Dussol, Sandrine Ménard, Margaux Papin, Maeva Boulée, Vérane Bard, Melvin Airaud, Daphna Fenel, Christine Moriscot, Frédérick Barreau, Marie Carriere

**Affiliations:** 1 Univ. Grenoble-Alpes, CEA, CNRS, Grenoble-INP, SyMMES-CIBEST, Grenoble, France; 2 IRSD, UMR 1220, INSERM, INRAE, Université de Toulouse, ENVT, Toulouse, France; 3 Integrated Structural Biology Grenoble (ISBG), UMS 3518, CNRS, CEA, Université Grenoble Alpes, Grenoble, France; 4 Univ. Grenoble-Alpes, CNRS, CEA, IBS, Grenoble, France

**Keywords:** crohn’s disease, inflammatory bowel disease, intestine, nanoplastics, polystyrene, toxicity

## Abstract

The daily use of plastic products leads to exposure of populations via ingestion, which may affect intestinal health. While a wealth of studies has documented the impact of micro- and nanoplastics (MNPLs) on models representing healthy human intestine, less is known about their impact on sensitive populations, particularly those at risk of developing inflammatory bowel diseases. This study explored the intestinal response to carboxylated and aminated polystyrene particles (50–500 nm PS-COOH and 50 nm PS-NH_2_) *in vivo* and *in vitro* in models of genetic susceptibility to Crohn’s disease. These models included nucleotide-binding oligomerization domain-2 wild-type and knock-out mice (Nod2^*WT*^ and Nod2^*KO*^, respectively) and co-cultures of Caco-2 and HT29-MTX cells, with Caco2 cells stably expressing either wild-type NOD2 or the NOD2^*1007fs*^ variant, which is strongly associated with Crohn’s disease. *In vitro,* the co-cultures were used either in a non-differentiated state (*i.e.*, 24 h post-seeding) or after differentiation into a tight, mucus-secreting epithelium. Results showed that *in vivo*, single oral administration of PS-COOH particles impaired the paracellular permeability of the ileum of Nod2^*WT*^ mice. Both PS-COOH and PS-NH_2_ reduced the number of goblet cells in Nod2^*WT*^ and Nod2^*KO*^ mice as well as Muc4 secretion in Nod2^*WT*^ mice, and PS-COOH reduced Muc4 secretion in Nod2^*KO*^ mice. Single oral administration of PS-COOH or PS-NH_2_ altered mRNA expression of genes involved in the production of antimicrobial peptide, inflammation, mucin secretion and endoplasmic reticulum stress pathways, in both Nod2^*WT*^ and Nod2^*KO*^ mice. *In vitro,* PS-COOH showed no impact in Caco2-NOD2^*1007fs*^/HT29-MTX and in Caco2-NOD2^*WT*^/HT29-MTX, whereas PS-NH_2_ induced a similar toxic response in both cell systems. The intensity of this response depended on the cells’ differentiation status and on the mode of exposure, *i.e.,* acute versus repeated. Overall, this study explored, for the first time, the response of models of genetic susceptibility to Crohn’s disease to exposure to PS-COOH and PS-NH_2_ nanoplastics (NPLs). The obtained data provide new insight into how populations at risk of developing CD respond to PS-COOH and PS-NH_2_ NPLs, i.e., new knowledge related to the impact of NPLs on this vulnerable population.

## 1 Introduction

The extensive use of plastic materials leads to contamination of the environment with micro- and nanoplastic particles (MNPLs), deriving from the release of plastic intentionally produced as micro- and nanoparticles (primary MNPLs) or from the degradation of bulk plastic (secondary MNPLs). Humans are mainly exposed to MNPLs through ingestion, as they have been detected in seafood, salt, bottled water, beer, honey and milk (Zhang et al., 2020; Toussaint et al., 2019). Such exposure can lead to ingestion of up to 53,864 particles per year (Danopoulos et al., 2020), including PS particles that are used in some yogurt containers, coffee cups, disposable cutlery and food containers (Europe, 2020). Although estimating human exposure using mass metrics is complex owing to particle size heterogeneity (Danopoulos et al., 2020), Senatirajah et al. estimated that approximately 0.1–5 g of MNPLs are ingested weekly (Senathirajah et al., 2021). The presence of MNPLs in human feces confirms human exposure, with higher quantities found in the faces of individuals with inflammatory bowel disease (IBD) than in those of healthy individuals (Schwabl et al., 2019; Yan et al., 2022; Zhang et al., 2021). Furthermore, a positive correlation has been reported between the concentration of MNPLs in stool samples and the severity of IBD (Yan et al., 2022). This result remains consistent regardless of drinking and dietary habits. The composition and size of MNPLs found in feces also differ with respect to the IBD status; however, PS MNPLs are observed in the feces of both populations (Yan et al., 2022). It is not documented whether these differences are related to the disease process itself, i.e., whether MNPLs potentially lead to the onset or exacerbation of IBD, or whether IBD patients exhibit higher MNPL retention in their intestines or ingest more MNPLs due to their dietary adaptations.

Although the literature on the impact of MNPL on the human intestine has grown considerably over the past 5 years, the mechanisms leading to their toxic impact are still not fully elucidated, particularly with regard to sensitive populations such as those with IBD or a genetic predisposition to developing these diseases. *In vivo*, mice orally exposed to PS particles exhibit typical features of Crohn’s disease (CD), including damaged intestinal crypts and villi, thinner intestinal walls and increased secretion of inflammatory mediators (Qiao et al., 2021; Xu et al., 2021). In mice models of colitis, PS particles exacerbate the symptoms, by triggering the release of inflammatory mediators, decreasing mucus secretion, increasing intestinal colon permeability and shortening its length (Luo et al., 2022; Zheng et al., 2021). *In vitro,* the accumulation of plain or COOH-functionalized PS particles in intestinal cells is well documented, with only mild signs of toxicity, which are observed only with the largest particles tested (with 500 nm and 1 µm in diameters) and at the highest concentrations tested (> 100 μg/mL) (Busch et al., 2021; Domenech et al., 2021; Domenech et al., 2020; Hesler et al., 2019; Stock et al., 2022; Stock et al., 2019; Xu et al., 2023; Zhang et al., 2022). Conversely, amine-functionalized PS particles (PS-NH_2_) show significant toxicity (see, for instance (Busch et al., 2021)), causing apoptosis, genotoxicity and inflammation, which has been attributed to lysosomal damage (Xia et al., 2008).

While the exact etiology of CD remains incompletely understood, it is known to involve genetic, immune and environmental factors. One of the most potent genetic mutations related to CD is the mutation of the nucleotide binding and oligomerization domain 2 (NOD2), which is a pattern-recognition receptor (PRR) also known as caspase activation and recruitment domain [CARD]15 and Nod-like receptor-C2. Between 30% and 40% of CD patients in North American and European populations have at least one NOD2 mutation, including the frequent p.Leu1007fsX1008 frameshift mutation (NOD2^*1007fs*^) (Eckmann and Karin, 2005; Economou et al., 2004). NOD2 regulates the interaction between the intestinal epithelium and immune cells and the microbiota by sensing bacterial muramyl dipeptide (MDP). This interaction leads to a host defense reaction characterized by the activation of the nuclear factor-κB (NF-κB) and mitogen-activated protein kinase (MAPK) signaling cascades, and the production of cytokine, chemokines and antimicrobial peptides, mucin secretion, as well as the activation of both innate and adaptive immune responses (Ferrand et al., 2019). NOD2 activation also stimulates autophagy via association with ATG16L1, thereby increasing intracellular bacterial clearance (Fritz et al., 2011). Furthermore, NOD2 activation by MDP negatively modulates the response of other PRRs to microbial stimuli, including Toll-like receptors (TLR), thereby contributing to the maintenance of a balanced intestinal response to pathogens (Strober et al., 2008). The p.Leu1007fsX1008 NOD2 mutation lies within the C-terminal leucine-rich repeat (LRR) domain, responsible for detecting pathogen- and damage-associated molecular patterns (PAMPs and DAMPs). Therefore, NOD2^*1007fs*^ fails to recognize MDP and consequently does not activate NF-κB and MAPK signaling cascades. It also does not mitigate pro-inflammatory cytokine responses mediated by TLRs and does not stimulate autophagy (Fritz et al., 2011). This altered response to bacteria, coupled to reduced ability to clear them, leads to chronic inflammation.

This study aimed to evaluate the impact of carboxylate- and amine-functionalized PS nanoplastic particles (NPLs, PS-COOH and PS-NH_2_) on intestinal models that are representative of populations with susceptibility to CD. Instead of using models of chemically induced colitis, which mimic the active phase of the disease (McKay et al., 1997), we used *in vivo* and *in vitro* models of genetic susceptibility to CD. These models were an *in vivo* model of mouse knocked-out (KO) for Nod2 (Nod2^*KO*^) and an *in vitro* model based on a co-culture of Caco-2 and HT29-MTX cells, which mimic a mucus-secreting intestinal epithelium, with genetically modified Caco-2 cells that permanently express either wild type NOD2 (NOD2^*WT*^) or NOD2^*1007fs*^. In addition to being a model of genetic susceptibility to CD, this model reproduces the intestinal epithelium phenotype, including tight junctions, expression of efflux pumps and microvilli (Dorier et al., 2019), as well as the protective function of mucus that protects intestinal epithelial cells from nanoparticles, including NPLs (Liévin-Le Moal and Servin, 2006). These models were used either in a proliferative, non-differentiated state, *i.e.,* exposed to NPLs 24 h after seeding, where they do not express tight junctions and microvilli, or after a 17-days post-confluence differentiation into an epithelium barrier. The impact of acute and repeated exposure to PS-COOH or PS-NH_2_ NPLs on cell viability, DNA integrity, the cells’ redox and inflammatory status were evaluated, as well as their capacity to accumulate PS-COOH or PS-NH_2_ NPLs.

*In vivo*, we report impact of both PS-COOH and PS-NH_2_ particles on paracellular intestinal permeability, villus length, number of Goblet and immune cells, while *in vitro* their impact is mild, with PS-NH_2_ being more cytotoxic than PS-COOH. PS-COOH NPLs heavily accumulate in cells where they locate mainly in the cytoplasm. Overall, this study brings valuable data on the impact of NPLs on models of genetic susceptibility to Crohn’s disease, both *in vitro* and *in vivo*, which had never been explored so far.

## 2 Materials and methods

### 2.1 Chemicals, particles and their characterization

Unless otherwise indicated, all chemicals were from Merck and were >99% pure. Carboxylated polystyrene NPLs (PS-COOH, Polybead® carboxylated, Polysciences), with 50, 100, 200 and 500 nm diameters and 50 nm aminated polystyrene NPLs (PS-NH_2_, fluorescent blue, Merck Sigma-Aldrich #L0780) were used throughout this study, except for fluorescence microscopy and quantification experiments, where Fluoresbrite® YG carboxylate PS microspheres (Polysciences) were used. All physico-chemical characterization methods and results, i.e., TEM images, primary diameter, size distribution, zeta potential in water and in cell culture medium, endotoxin contamination are reported in Supplementary Material Section 1 (Supplementary Table S1; Supplementary Figure S1).

### 2.2 *In vivo* experiments

The *in vivo* experiments were performed following strict guidelines for animal experimentation. C57BL/6 wild-type (Nod2^*WT*^) and Nod2^*KO*^ mice (Barreau et al., 2007) were housed in specific pathogen-free conditions and monitored every 6 months in accordance with the full set of FELASA recommendations. They were generated in the US006/CREFE animal facility, housed in pathogen-free conditions and maintained under 12 h light–dark cycles with free access to food and water. The animal care and ethics committee of US006/CREFE (APAFIS #34331–2021121309394360 v4) and 34067_BE35/2022 approved all experimental procedures, which were conducted following the guide for care and use of laboratory animals of the European Council. C57BL/6 Nod2^*WT*^ and Nod2^*KO*^ mice (Barreau et al., 2007) were exposed to a single oral gavage of 1.25 mg of PS-COOH or PS-NH_2_ particles, then sacrificed 24 h later. At least 8 mice per group were used, from 3 independent experiments. Exposure to PS-COOH and exposure to PS-NH_2_ were performed in separate experiments.

Our aim was to expose mice to a realistic dose of MNPLs, as calculated based on current estimations at the time when the experiments were conducted. Considering weekly human ingestion of MNPLs being up to 5 g (Senathirajah et al., 2021), *i.e.,* 0.2–10 mg of MNPLs/kg body weight (b.w.)/week for a 70 kg adult, it corresponds to exposing mice to 2.5–125 mg MNPL/kg b.w./week according to the FDA guidance for animal-to-human dose conversion (FDA, 2005). Considering that the weight of an adult mouse is 30 g, this corresponds to ingestion of 0.01–0.54 mg MNPLs in 1 day. Therefore, exposing mice to 1.25 mg MNPLs can be considered as a realistic doses, since it is 2.5-fold the highest range of human exposure and since some intestinal regions may be exposed to a higher dose than the average due to intestinal peristaltism. Intestinal samples were frozen at −80 °C for RT-qPCR and ELISA. For histology, biopsies from at least 4 mice from 2 independent experiments were collected, fixed in 10% formalin/ 4% formaldehyde for 48 h, then in 70% ethanol, included in paraffin and cut as 5 µm-thick sections before hematoxylin/ eosin coloration. Images were acquired with Panoramic Scan Flash III and analyzed with CaseViewer. A minimum of 8 villi per sample were measured and the number of goblet cells and immune cells per 100 µm of villi was counted. Intestinal passage of fluorescein across small intestine and colon was assessed in Ussing chambers (Physiologic Instruments, San Diego, CA, USA) (Riba et al., 2018), in at least 8 mice per group from 3 independent experiments. To that, tissues were bathed 1 h with oxygenated thermostated Ringer solution. Fluorescein Sodium Salt 40 μg/ml (376 kDa; Sigma) was added to the mucosal compartment and intestinal passage was determined by measuring fluorescence intensity at λexc./λem. 485 nm/525 nm using an automatic varioskan microplate reader.

### 2.3 Cell culture and exposure

HT29-MTX cells were a generous gift of T. Lesuffleur (INSERM, Paris). Caco2 cells (ATCC HTB37) were transduced with a lentivirus expressing the NOD2 gene, either wild-type or carrying the 1007fs frameshift mutation, i.e., Caco2-NOD2^*WT*^ and Caco2-NOD2^*1007fs*^, respectively. NOD2 expression and functionality in these cell lines was assessed as described in Supplementary Material Section 2 (Supplementary Figure S2). Caco2-NOD2^*WT*^, Caco2-NOD2^*1007fs*^ and HT29-MTX were grown in DMEM/glutamax containing 1% penicillin/streptomycin, 1% non-essential amino acids and 10% fetal bovine serum (FBS). They were maintained at 37 °C in a humidified 5% CO_2_ incubator and passed twice a week with trypsin-EDTA. Caco2-NOD2^*WT*^ and Caco2-NOD2^*1007fs*^ were seeded with HT29-MTX at a ratio of 90% Caco2 and 10% HT29-MTX cells, at 80000 cells per cm^2^. They were exposed to NPLs either 24 h after seeding (non-differentiated cells, which do not express tight junction and microvilli and are still proliferative), , or after growing post-confluence for 17 days, changing the medium twice or three times a week, which allows Caco2 to differentiate into mature, polarized, enterocyte-like cells with tight junctions and microvilli, as in the intestine (Dorier et al., 2019; Walter et al., 1996). When differentiated, they are considered to be more representative of the intestinal epithelium morphology and function, especially as they express tight junctions and xenobiotic efflux pumps. Still, the apical brush border of intestinal epithelium has been shown to be able to undergo endocytosis but to limit the trafficking of endosomes (Hanse et al., 2009). Since nanoparticles are known to be internalized in cells via endocytosis, the apical brush border of Caco2 cells, when differentiated, may hamper nanoparticle trafficking through the cells, which can explain their reduced sensitivity to nanoparticles’ toxic impact as compared to non-differentiated Caco2 cells, as observed for example for graphene oxide sheets (Kucki et al., 2017). Differentiated cells were exposed to PS-COOH or PS-NH_2_ particles either acutely (single exposure for 24 h) or repeatedly, i.e., once a day for four consecutive days to ¼ the PS-COOH or PS-NH_2_ concentration used in the acute exposure condition. The concentrations to which cells were exposed to NPLs ranged between 3.1 and 100 μg/mL. They are consistent with those used by others, making possible the comparison of results obtained here with those of the literature. Considering that humans may ingest up to 5 g of MNPLs per week (Senathirajah et al., 2021) and that the estimated surface of human intestine is 32 m^2^ (Helander and Fändriks, 2014), one can calculate human intestinal exposure to be ∼2.2 µg MNPs per cm^2^ of intestine. Concentrations ranging from 3.1 to 100 μg/mL correspond to 1–33 μg/cm^2^ (for dose conversion from µg/mL to µg/cm^2^, see (Bard et al., 2026; Boulée et al., 2026)). These concentrations are in the range of the estimated human daily exposure; therefore, they can be considered realistic.

### 2.4 Intracellular accumulation of PS-COOH NPLs

Uptake of fluorescent PS-COOH particles was imaged using confocal microscopy, on cells exposed to 50 μg/mL yellow-green fluorescent PS-COOH particles, then washed three times with PBS and fixed for 15 min with 4% paraformaldehyde at room temperature (RT). After washing with PBS, their actin cytoskeleton and nucleus were stained for 30 min at RT with 0.4 µM Phalloidin Atto-440 and 10 μg/mL DAPI, then slides were mounted with Prolong Gold (ThermoFisher Scientific). Images were recorded on a Zeiss LSM880 confocal microscope equipped with a fast AiryScan detector. PS-COOH intracellular distribution was also imaged by TEM. Cells exposed to 200 nm PS-COOH were washed with 0.1 M PHEM buffer, fixed for 1 h with 2% formaldehyde/0.2% glutaraldehyde and post-fixed with 1% osmium tetroxide containing 1% uranyl acetate. They were dehydrated with a graded series of ethanol and embedded in Epon, cut to 80 nm ultrathin sections and observed using a JEOL 1200EX TEM operating at 80 kV. For PS-COOH NPL uptake quantification, cells were seeded in black 96-well plates and exposed to fluorescent PS-COOH particles, using five wells per condition. After three washes with PBS, wells were filled with 100 µL of PBS and fluorescence was measured at λabs/λem 480/530 nm using a Spectramax ID3 spectrofluorimeter (Molecular Devices). The experiment was repeated three times (n = 3), resulting in 15 experimental values. In the acute exposure condition, PS-COOH accumulation was also analysed via flow cytometry with a CytoFlex S (Beckman), using the percentage of fluorescent cells and the mean fluorescence intensity (MFI) of gated cells as criteria. Unfortunately, PS-NH_2_ intracellular accumulation could not be visualized or quantified using these techniques. Indeed, although fluorescent blue, their fluorescence emission was too low to be detected. Fluorescence measurement using a Spectramax ID3 spectrofluorimeter (Molecular Devices) of a serial dilution of these NPLs led to significant detection down to 1 μg/mL.

### 2.5 Cytotoxicity and impact on cell barrier integrity

PS-COOH and PS-NH_2_ cytotoxicity were evaluated using the WST-1 assay, on cells seeded in 96-well plates and exposed to 6.25–100 μg/mL NPLs. In each experiment, 5 replicates of each concentration were used (5 separate wells), and the whole experiment was repeated three times independently (n = 3), resulting 15 experimental values. After exposure, the medium was replaced by 100 µL of a 10-fold diluted WST-1 solution (Merck), and incubated 45 min at 37 °C. Absorbance at 450 nm was measured using a Spectramax ID3 spectrofluorimeter (Molecular Devices). Half-maximum inhibitory concentrations (IC50), *i.e*., NPL concentrations leading to 50% cell viability loss, were calculated using nonlinear regression in GraphPad Prism (v7.05). The used parameters were variable slope (four parameters) and least square (ordinary) fit.

### 2.6 Oxidative stress and DNA damage

Dihydrorhodamine 123 (DHR123) was used to estimate reactive oxygen species (ROS) content in cells after exposure to NPLs. Prior to exposure to PS-COOH and PS-NH_2_ NPLs, cells were incubated for 45 min at 37 °C with 1 µM DHR123, then washed with PBS. Then, they were exposed to 50 μg/mL PS-COOH or 6.2 μg/mL PS-NH_2_, applied to 5 separate wells. Rhodamine 123 fluorescence was monitored immediately after exposure (t0), then after 30 min, 1 h, 2 h, 4 h, 6 h and 24 h of exposure, using a Spectramax ID3 spectrofluorimeter (Molecular Devices). Fluorescence was normalized with respect to the fluorescence at t0. The whole experiment was repeated three times independently (n = 3), resulting in 15 experimental values (5 technical replicates in each of the 3 biological replicates).

DNA strand breaks were quantified using the alkaline comet assay in its simple version without Fpg (Azqueta and Dusinska, 2015). Cells were exposed to 25 or 50 μg/mL of PS-COOH or to 3.1 or 6.2 μg/mL of PS-NH_2_. These concentrations were applied in three separate wells per condition. The whole exposure was performed once, but each of the three separate wells was analyzed in an independent comet assay (n = 3 independent experiments). After exposure, cells were harvested and included in 1% low melting point agarose, deposited as two 70 µL gels on slides pre-coated with agarose, then allowed to solidify on ice. As positive controls, cells were exposed for 24 h to 30 μg/mL methyl methanesulfonate (MMS). Cells in gels were lyzed for 1 h at room temperature in 2.5 M NaCl, 10 mM Tris.base, 0.1 M EDTA and 1% Triton, pH 10, then rinsed three times with PBS and immersed in cold electrophoresis buffer (NaOH 0.3 M, EDTA 1 mM, pH > 13) for 40 min. Then, the electrophoresis was performed at 1.2 V/cm for 30 min. Slides were washed twice for 10 min in PBS and stained with GelRed^TM^ (Biotium). Fifty comets per gel were scored using Comet Assay IV (Perceptive Instruments). This resulted in a total of 6 values per condition (2 gels per condition for each comet experiment, 3 independent comet experiments).

### 2.7 ELISA

*In vivo* samples from at least 4 mice per group from 3 independent experiments were lysed with 1 ml of RIPA buffer (Merck Sigma Aldrich) containing proteases inhibitor (cOmplete™, Mini, EDTA-free, Roche) using Lysing Matrix D tubes (Fastprep) and a Precellys 24 Touch homogenizer. After centrifugation for 10 min at 1,600 × *g* at 4 °C, protein concentrations were measured using Pierce™ BCA Assay (Thermo scientific). Mucin-4 (MUC4) and Regenerating islet-derived protein 3γ (REG3γ) levels in the ileum were determined using ELISA kits following the manufacturer’s instructions (Assay Genie). *In vitro*, the release of CXCL8 by cells exposed to NPLs was quantified using BD Biosciences ELISA assay following the manufacturer’s instructions. To that, cells were exposed to 25 μg/mL or 50 μg/mL PS-COOH or to 3.1 μg/mL or 6.2 μg/mL PS-NH_2_, 3 wells being exposed for each concentration. The whole experiment was repeated three times (n = 3), resulting in 9 experimental values.

### 2.8 Reverse transcriptase-quantitative polymerase chain reaction (RT-qPCR)

RNA was extracted using GeneElute kit (Merck) following the manufacturer’s instructions, with mechanical lysis via a tissue lyzer (Qiagen) for *in vivo* samples or with lysis by pipetting up and down several times in lysis buffer for *in vitro* cells. RNA concentration and purity was evaluated using a Nanodrop, via measuring Abs. 260/Abs. 280 and Abs. 230/Abs. 280 ratios. RNA was reverse-transcribed using superscript III. Quantitative PCR was performed with Takyon No ROX SYBR MasterMix blue dTTP (Eurogentec), with cycling parameters 95 °C, 5 min; 40 cycles of (95 °C, 15 s; 55 °C, 20s; 72 °C, 40 s); then 95 °C, 1 min; 55 °C, 30 s and 95 °C, 30s for the dissociation curve. Primer sequences are reported in Supplementary Material Section 4 (Supplementary Table S2). Reference genes were validated using BestKeeper (Pfaffl et al., 2004) and results were analysed using the ΔΔCq method with REST2009 (Pfaffl et al., 2002). In the *in vivo* experiment, samples from 4 exposed mice (n = 4) were analyzed. Their extracted RNA was loaded in duplicate in the qPCR plate. In the *in vitro* experiment, three independent wells were exposed to each NPL concentration. The experiment was not repeated. Extracted RNAs were loaded twice in the qPCR plate, resulting in 8 (*in vivo*) or 6 (*in vitro*) experimental values.

### 2.9 Statistical analysis

In the *in vivo* experiments, statistical analyses were performed using GraphPad Prism version 10.00 (GraphPad software, San Diego, CA). All datasets were analyzed with ROUT test to exclude outliers. Multigroup comparisons were performed using a 1-way analysis of variance followed by a Bonferroni correction for multiple comparisons. Two-group comparisons were performed using unpaired t-tests, assuming that data followed a Gaussian distribution, which was verified using a Kolmogorov-Smirnov test. A threshold of P < 0.05 was set for statistical significance. All P values indicated are 2-tailed. Results are expressed as mean +/- sem.

In all *in vitro* experiments except RT-qPCR, as data did not follow a normal distribution and variances were not homogeneous, non-parametric statistical tests were performed, *i.e*., Kruskal-Wallis tests, followed by the Dunn’s multiple comparison tests, using GraphPad Prism (version 7.05). Results are reported as box and whiskers plots, showing the minimum value, lower quartile, median, upper quartile and maximum value. Results were considered statistically significant (* or #) when the p-value was less than 0.05. For RT-qPCR experiments, data were analyzed using REST2009, which uses randomization and bootstrapping methods (Pfaffl et al., 2002).

## 3 Results

### 3.1 *In vivo* impact of PS-COOH and PS-NH_2_ on the intestinal barrier function

**FIGURE 1:**
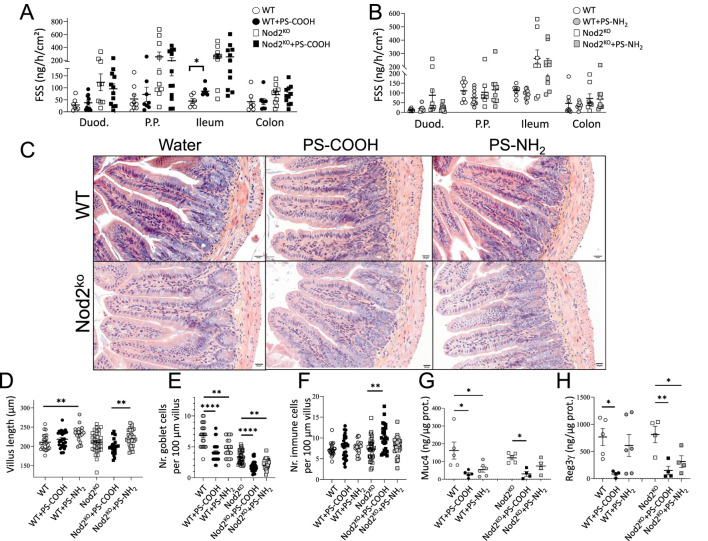
*In vivo* impact of PS-COOH and PS-NH_2_ on Nod2^WT^ and Nod2^KO^ mice. Duodenum (Duod.), Peyer’s patches (P.P), ileum and colon permeability after a single exposure to 1.25 mg of PS-COOH or PS-NH_2_ particles, assessed in at least eight mice per group (from three independent experiments), either Nod2^WT^ **(A)** or Nod2^KO^ **(B)**. Hematoxylin/eosin staining of ileum thin sections from at least four mice per group, from two independent experiments, either Nod2^WT^ (WT) or Nod2^KO^ and either non exposed (Water) or exposed to PS-COOH or PS-NH_2_ NPLs **(C)**. These histological sections made possible the measurement of villus length **(D)**, of goblet cells number **(E)** and immune cells number **(F)**. ELISA quantification of mucin 4 (Muc4) **(G)** and Reg3g **(H)** protein expression in the ileum, measured in at least four animals per group, from three independent experiments.

The response of Nod2^*WT*^ and Nod2^*KO*^ mice was evaluated 24 h after single oral administration of 1.25 mg of 50-nm-diameter PS-COOH and PS-NH_2_ NPLs. First, their impact on paracellular permeability was explored. Nod2^*KO*^ mice showed higher basal paracellular permeability compared to WT mice, and this difference was statistically significant (Supplementary Figure S3). Exposure to PS-COOH significantly increased paracellular permeability in WT mice, but not in Nod2^*KO*^ mice, and only in the ileum (Figure 1A). Exposure to PS-NH_2_ had no effect on paracellular permeability (Figure 1B).

Macroscopically, the intestinal morphology did not look impacted by any treatment, whatever the mouse model. It showed a dense network of well-organized villi with wall thickness comparable to those of unexposed animals (Figure 1C). Nevertheless, significant increase in mean villus length was observed in WT mice exposed to PS-NH_2_, compared with unexposed WT mice, and in Nod2^*KO*^ mice exposed to PS-NH_2_, compared to mice exposed to PS-COOH (Figure 1D). The number of goblet cell was reduced in both mouse models exposed to both PS-COOH and PS-NH_2_ particles, compared to their respective controls (Figure 1E). Moreover, the number of immune cells was significantly increased in Nod2^*KO*^ mouse exposed to PS-COOH, compared to control, but not in mice exposed to PS-NH_2_ (Figure 1F).

The impact of PS-COOH and PS-NH_2_ exposure on some molecular pathways related to IBD were explored by RT-qPCR, *i.e.*, inflammation, endoplasmic reticulum stress, mucus production and antimicrobial peptide secretion, as reported in Supplementary Material Section 3 (Supplementary Table S3). PS-COOH decreased the mRNA expression of some inflammation markers, i.e., Il1-β in WT mice (x 0.58) and Ifn-γ in both WT and Nod2^*KO*^ mice (x 0.64 and x 0.50, respectively), while it increased Tnf-α (x 3.42) and Il-12 (x 5.99) mRNA expression in Nod2^*KO*^ mice. PS-NH_2_ only decreased mRNA expression of Tnf-α in Nod2^*KO*^ mice (x 0.59) (Supplementary Table S3). In the mucin secretion pathway, PS-COOH induced a drop in Muc4 and Tff3 mRNA expression in WT mice (x 0.68 and x 0.59, respectively), and of Muc2 and Muc4 in Nod2^*KO*^ mice (x 0.49 and x 0.59, respectively) (Supplementary Table S3). It also affected the antimicrobial peptide pathway, as PS-COOH exposure decreased Mmp7, Reg3β and Reg3γ mRNA expression in both WT (x 0.45, x 0.26, x 0.24, respectively) and Nod2^*KO*^ (x 0.24, x 0.39, x 0.38, respectively) mice, and Lyz1p and AllCrypt mRNA expression in Nod2^*KO*^ mice. PS-NH_2_ induced a drop in Reg3β mRNA expression in WT and Nod2^*KO*^ mice (x 0.66 and x 0.44, respectively), and a decrease of Mmp7 and Lyz1p in Nod2^*KO*^ mice (Supplementary Table S3). The unfolded protein response pathways, which is triggered upon endoplasmic reticulum stress, was also affected. PS-COOH decreased Atf4, Bip, Grp94 and Xbp1 mRNA expressions in WT mice (x 0.63, x 0.59, x 0.47, x 0.49, respectively) and of Atf4, Atf6, Grp94 and Xbp1 in Nod2^*KO*^ mice (x 0.54, x 0.59, x 0.76, x 0.46, respectively). It increased mRNA expression of Chop in Nod2^*KO*^ mice (x 1.48). PS-NH_2_ decreased Bip, Grp94 and total Xbp1 mRNA expression in WT mice (x 0.59, x 0.78, x 0.85, respectively), and of Atf4 and Bip in Nod2^*KO*^ mice (x 0.64 and x 0.56, respectively) (Supplementary Table S3). Finally, no impact of PS-COOH and PS-NH_2_ particles was observed on tight junctions in WT mice. However, in Nod2^*KO*^ mice, PS-COOH decreased Cldn2 mRNA expression, while PS-NH_2_ increased Ocln and Zo1 mRNA expression. The mRNA expression of Mlck, involved in intestinal permeability, was decreased in the ileum of WT mice exposed to PS-COOH (x 0.65) and of Nod2^*KO*^ mice exposed to PS-NH_2_ (x 0.45) (Supplementary Table S3). Noteworthy, evaluating the modulation of mRNA expression level cannot be considered as a proof of protein function modulation, as the mRNA level does not necessarily reflect the protein function. Therefore, without any confirmation at the protein or at the functional level, such results should be interpreted with caution. As a confirmation, impact on mucin and antimicrobial peptide secretion was also evaluated at the protein level, which revealed decreased MUC4 protein level in the ileum in both WT and Nod2^*KO*^ mice exposed to PS-COOH, and in Nod2^*KO*^ mice exposed to PS-NH_2_ (Figure 1G). REG3γ protein level was also decreased in the ileum in WT mice exposed to PS-COOH and in Nod2^*KO*^ mice exposed to both NPLs, compared to the respective unexposed mice (Figure 1H).

Overall, depending on the exposure condition, these results indicate that exposure to PS-COOH and/or PS-NH_2_ cause a mild intestinal inflammation, affect mucus and AMP secretion and would disturb endoplasmic reticulum homeostasis.

### 3.2 Impact of PS-COOH and PS-NH_2_ in *in vitro* models of genetic susceptibility to Crohn’s disease

#### 3.2.1 Impact of PS-COOH and PS-NH_2_ in differentiated Caco2-NOD2/HT29-MTX cells

To mimic human intestine with genetic susceptibility to CD, *in vitro*, we used Caco-2 cells that had been transduced to permanently express wild-type or mutated NOD2, *i.e.*, Caco2-NOD2^*WT*^ and Caco2-NOD2^*1007fs*^. To enhance the representativeness of these *in vitro* models of the human ileum, these cells were co-cultured with HT29-MTX cells that permanently secrete high amounts of mucus (Lesuffleur et al., 1990). Such co-cultures were grown for 17 days post-confluence to allow Caco-2 cell differentiation into enterocyte-like cells, so that the co-culture reproduced an intestinal epithelium with tight junctions, microvilli and mucus secretion (Dorier et al., 2019; Walter et al., 1996). Then, they were exposed for 24 h to PS-COOH and PS-NH_2_.

**FIGURE 2:**
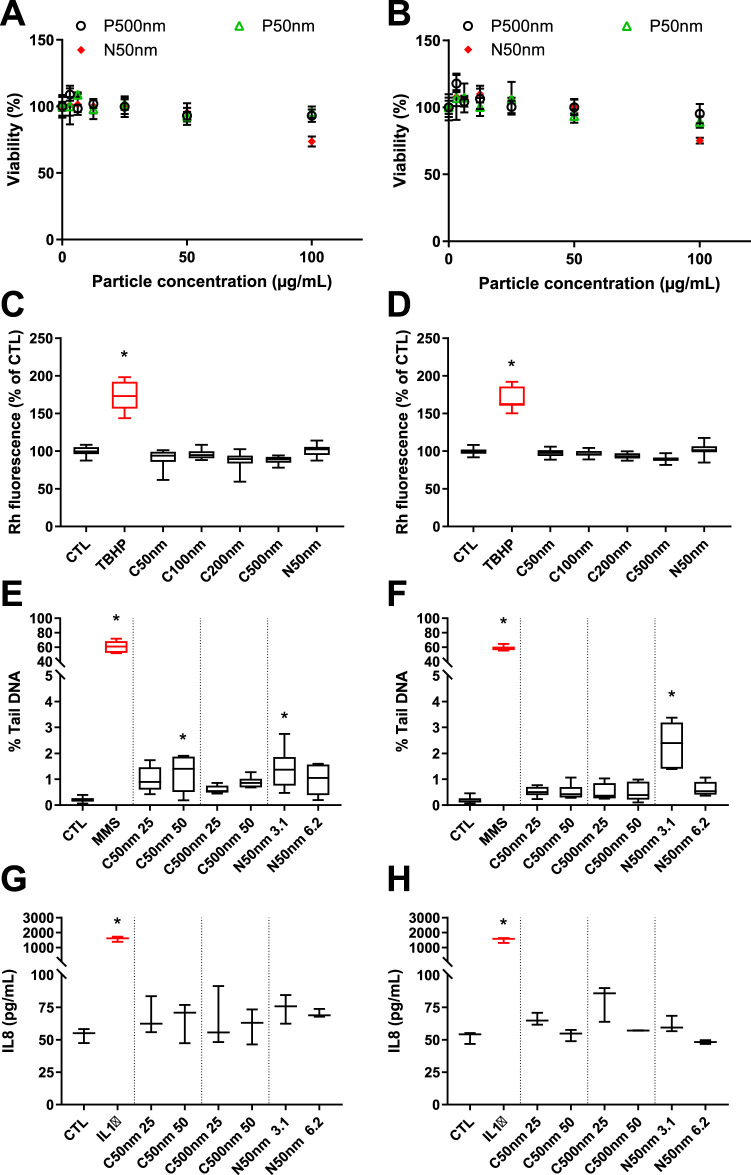
PS-COOH and PS-NH_2_ NPLs toxicity upon acute exposure of differentiated Caco2-NOD2^*WT*^ and Caco2-NOD2^*1007fs*^/HT29-MTX cells. Cells were grown 17 days post-confluence, then exposed for 24 h to PS particles. Viability was assessed via the WST-1 assay **(A,B)**, intracellular accumulation of reactive oxygen species was probed via the DHR123 assay **(C,D)**, genotoxicity via the comet assay **(E,F)** and IL-8 secretion was quantified via ELISA **(G,H)**, on Caco2-NOD2^*WT*^/HT29-MTX cells **(A,C,E,G)** or on Caco2-NOD2^*1007fs*^/HT29-MTX cells **(B,D,F,H)**. In the DHR123 assay cells were exposed to 50 μg/mL PS-COOH particles or to 6.2 μg/mL of PS-NH_2_ particles. In the comet and IL-8/CXCL-8 assays, cells were exposed to 25 μg/mL or 50 μg/mL of PS-COOH or to 3.1 μg/mL or 6.2 μg/mL of PS-NH_2_. *p < 0.05, exposed vs. CTL, n = 3 independent experiments.

PS-COOH and PS-NH_2_ showed low impact on cell viability in both cell models. Only cells exposed at the highest tested concentration of PS-NH_2_, showed ∼25% of cell viability loss (Figure 2A-B). As viability loss was <50%, IC50 could not be determined. The TEER was also measured after 24 h of exposure to 50 nm or 500 nm PS-COOH or to 50 nm PS-NH_2_ and no statistically significant decrease in TEER was observed (Supplementary Figure S4). Neither PS-COOH nor PS-NH2 caused significant impact on ROS intracellular levels (Figure 2C-D). Conversely, a statistically significant increase in DNA strand break level was observed in Caco2-NOD2^*WT*^/HT29-MTX exposed to 50 μg/mL 50 nm PS-COOH, and in both Caco2-NOD2^*WT*^/HT29-MTX and Caco2-NOD2^*1007fs*^/HT29-MTX exposed to 3.1 μg/mL 50 nm PS-NH_2_. Both these increases were mild, reaching only 1.5%–2% Tail DNA while the % Tail DNA in unexposed cells was ∼0.2% and positive control reached ∼60% Tail DNA (Figure 2E-F). This represented a 6 ± 4, 10 ± 8 and 18 ± 13-fold increase in % Tail DNA, respectively (exposed cells *vs*. unexposed cells). This increase was mild and the variability of results was high, which questions the physiological significance of this result. Finally, such exposure did not significantly increase CXCL8 secretion (Figure 2G-H).

**FIGURE 3:**
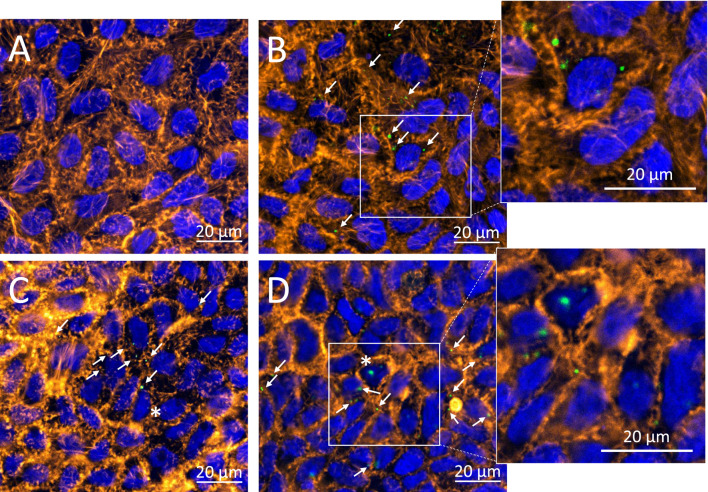
Cellular uptake of PS-COOH particles in differentiated Caco2-NOD2/HT29-MTX cells. Confocal images of Caco2-NOD2^*WT*^/HT29-MTX **(A–D)** cells, either not exposed **(A)** or exposed to 50 μg/mL of 50 nm **(B)** 100 nm **(C)** or 200 nm **(D)** yellow-green fluorescent PS-COOH for 24 h. Blue: DAPI (cell nuclei); orange: phalloïdin-Atto440 (actin network); green: fluorescent PS-COOH particles. Similar confocal images of Caco2-NOD2^1007fs^/HT29-MTX cells are provided in Supplementary Figure S5. Particles are located mainly in the cytoplasm (arrows), rarely in the nucleus (asterisk). Apparent intra-nuclear localization of PS-COOH (asterisk) is likely attributable to fluorescence bleed-through across z-stacks, as discussed in Supplementary Figure S6. Scale = 20 μm.

Whatever their diameter, PS-COOH accumulated in both cell models, as probed via confocal microscopy. Inside cells, they mainly distributed in the cytoplasm (Figure 3 and Supplementary Figure S5, arrows, in Caco2-NOD2^*WT*^/HT29-MTX and in Caco2-NOD2^*1007fs*^/HT29-MTX, respectively) and rarely in the nucleus (Figure 3 and Supplementary Figure S5, stars, in Caco2-NOD2^*WT*^/HT29-MTX and in Caco2-NOD2^*1007fs*^/HT29-MTX, respectively). Nevertheless, these fluorescent PS-COOH were so bright that they shined in several consecutive stacks in confocal microscopy (Supplementary Figure S6), suggesting that intra-nuclear localization could be an artifact. When quantifying PS-COOH accumulation via cell fluorescence measurement, for each particle size, a significant concentration-dependent increase was observed (Supplementary Figure S7). Intracellular accumulation was similar in Caco2-NOD2^*WT*^/HT29-MTX (Supplementary Figure S7A) and in Caco2-NOD2^*1007fs*^/HT29-MTX (Supplementary Figure S7B). Unfortunately, PS-NH_2_ intracellular accumulation could not be evaluated, due to the low fluorescence emission of the blue fluorescent PS-NH_2_ used in this study.

**FIGURE 4:**
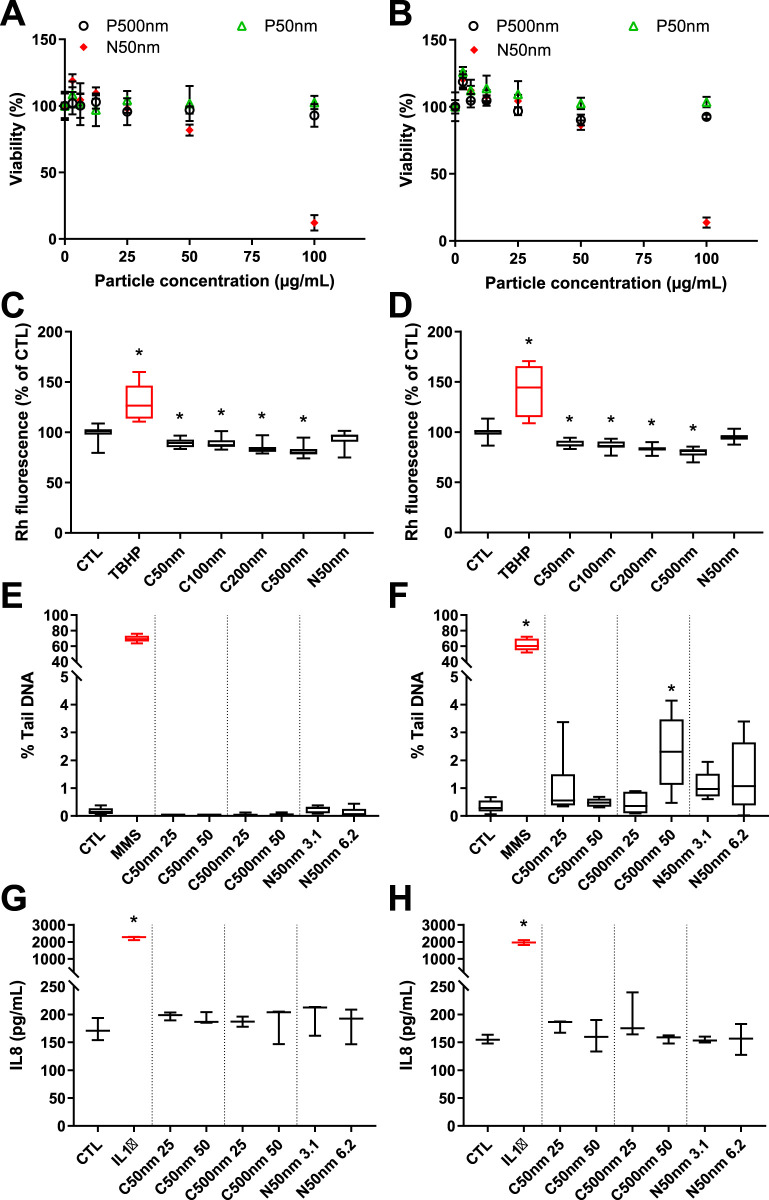
PS-COOH and PS-NH_2_ NPLs toxicity upon repeated exposure of differentiated Caco2-NOD2/HT29-MTX cells. Cells were grown 17 days post-confluence and then exposed daily for 4 days to PS particles, at ¼ the concentration used in acute exposure assays, *i.e.,* in the DHR123 assay 12.5 μg/mL of PS-COOH per day for 4 days to achieve 50 μg/mL of PS-COOH, or 1.55 μg/mL.day of PS-NH_2_ for 4 days to achieve 6.2 μg/mL. In the comet and IL-8 assays cells were exposed to 6.25 or 12.5 μg/mL of PS-COOH per day for 4 days to achieve 25 or 50 μg/mL of PS-COOH, respectively, and 0.775 or 1.55 μg/mL of PS-NH_2_ per day for 4 days to achieve 3.1 or 6.2 μg/mL of PS-NH_2_, respectively. Viability was assessed via the WST-1 assay **(A,B)**, intracellular accumulation of reactive oxygen species was probed via the DHR123 assay **(C,D)**, genotoxicity via the comet assay **(E,F)** and IL-8 secretion was quantified via ELISA **(G,H)**, on Caco2-NOD2^*WT*^/HT29-MTX cells **(A,C,E,G)** or on Caco2-NOD2^*1007fs*^/HT29-MTX cells **(B,D,F,H)**. In comet and IL-8/CxCL-8 assays, cells were exposed to 25 μg/mL or 50 μg/mL of PS-COOH or to 3.1 μg/mL or 6.2 μg/mL of PS-NH_2_. *p < 0.05, exposed vs. CTL, n = 3 independent experiments.

Next, to explore the influence of the dose rate on the cellular response to these NPLs, differentiated Caco2-NOD2/HT29-MTX cells were exposed repeatedly to PS-COOH and PS-NH_2_ particles once a day for four consecutive days, simulating chronic exposure. The concentration applied to the cells each day was one fourth of that applied in the corresponding acute exposure condition. Again, no significant cytotoxicity was observed for PS-COOH particles, while PS-NH_2_ significantly affected cell viability at the highest tested concentration, i.e., 100 μg/mL (*i.e*., 25 μg/mL per day for four consecutive days). The response was more intense response than that of acute exposure, with >90% cell viability loss (Figure 4A-B). IC50 for PS-NH_2_ was 62.57 ± 3.19 μg/mL (R^2^ = 0.9691) and 63.65 ± 2.95 μg/mL (R^2^ = 0.9753) in Caco2-NOD2^*WT*^/HT29-MTX and in Caco2-NOD2^*1007fs*^/HT29-MTX, respectively. PS-COOH slightly decreased intracellular ROS content in both cell models, while PS-NH_2_ showed no impact on ROS levels (Figure 4C-D). Such low impact might suggest that cells successfully respond to PS-COOH and PS-NH_2_ exposure in order to restore redox homeostasis. Only exposure to 50 μg/mL (i.e., 12.5 μg/mL per day for four consecutive days) of 500 nm PS-COOH caused a statistically significant increase in DNA strand break level (Figure 4E-F). This represented an 8 ± 4-fold increase in % Tail DNA (exposed cells vs. unexposed cells). Again, although statistically significant, the variability of results was high, which questions their physiological significance. No increase of CXCL8 secretion was observed (Figure 4G-H).

As in the acute exposure mode, PS-COOH accumulated in cells. Their accumulation level increased with the exposure concentration, and was similar in Caco2-NOD2^*WT*^/HT29-MTX and in Caco2-NOD2^*1007fs*^/HT29-MTX (Supplementary Figure S8). Via confocal microscopy, PS-COOH distributed mainly in the cell cytoplasm (Supplementary Figure S9, arrows) and sometimes in the nucleus (Supplementary Figure S9, stars).

These results indicate that PS-COOH heavily accumulate in these differentiated cells, that PS-NH_2_ are more toxic than PS-COOH, although their toxicity upon short-term exposure is mild, and that repeated exposure leads to stronger impact of PS-NH_2_ on cell viability compared to acute exposure. They also suggest that differentiated cells respond to PS-COOH and PS-NH_2_ exposure by restoring homeostasis, generating low-grade DNA damage or modulating the redox response, without causing any major toxic effects.

#### 3.2.2 Impact of PS-COOH and PS-NH_2_ in non-differentiated Caco2-NOD2/HT29-MTX cells

##### 3.2.2.1 Cell response to PS-COOH and PS-NH_2_ exposure

Post-confluent, differentiated co-cultures of Caco-2/HT29-MTX cells are reported to be less sensitive to toxicants than non-differentiated Caco-2/HT29-MTX cells (Ude et al., 2017). Therefore, we repeated the PS-COOH and PS-NH_2_ toxicity evaluation in non-differentiated Caco2-NOD2^*WT*^ and Caco2-NOD2^*1007fs*^ co-cultured with HT29-MTX cells, to decipher if this more sensitive cell system would be more prone to reproduce the effect of PS-COOH and PS-NH_2_ that we had observed *in vivo*.

**FIGURE 5:**
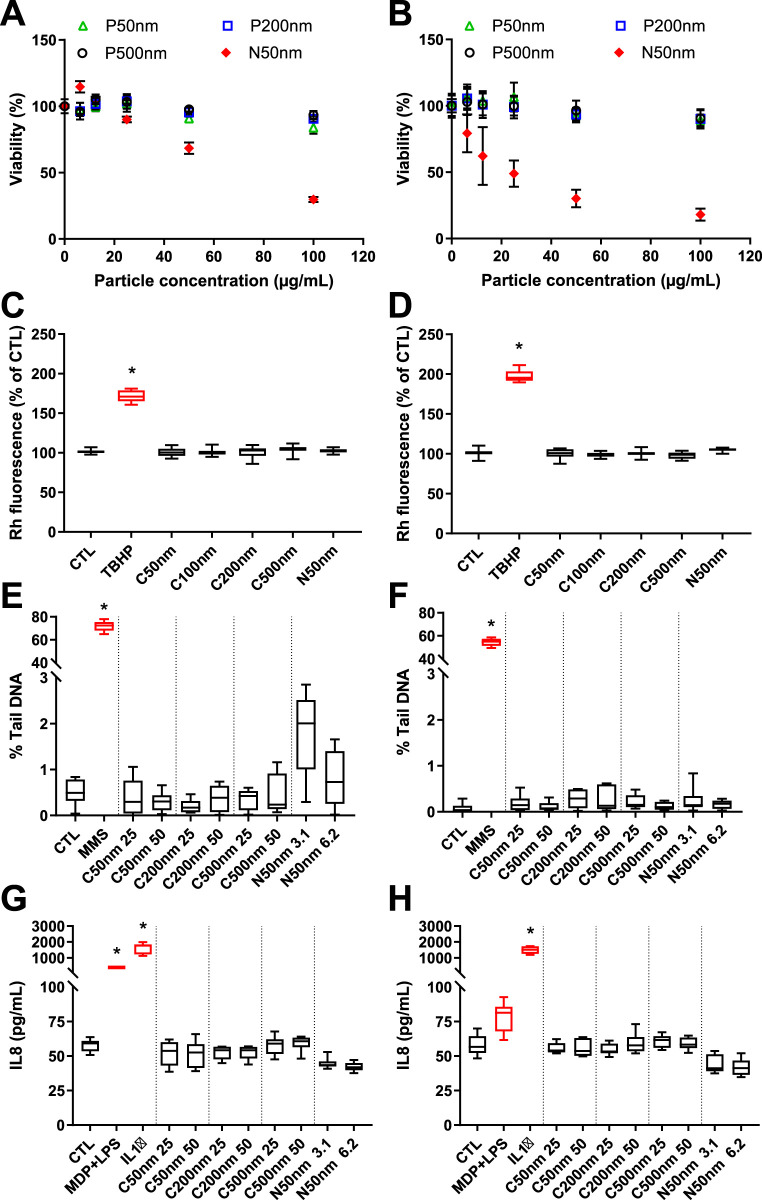
Impact of PS-COOH and PS-NH_2_ NPLs upon acute exposure of non-differentiated Caco2-NOD2/HT29-MTX cells. Cell viability was assessed via the WST-1 assay **(A,B)**, reactive oxygen species were quantified using the DHR123 probe **(C,D)**, genotoxicity was assessed via the alkaline comet assay **(E,F)** and IL-8 release by ELISA **(G,H)** on a Caco2-NOD2^*WT*^/HT29-MTX co-culture **(A,C,E,G)** or on Caco2-NOD2^*1007fs*^/HT29-MTX cells **(B,D,F,H)**. In the DHR123 assay, cells were exposed to 50 μg/mL of PS-COOH or 12.5 μg/mL of PS-NH_2_ particles. In the comet assay and IL-8 assay, cells were exposed to 25 μg/mL or 50 μg/mL of PS-COOH or to 3.1 μg/mL or 6.2 μg/mL of PS-NH_2_. Positive controls were tert-butyl hydroperoxide (1 mM) in the DHR123 assay, MMS (30 μg/mL, 24 h) in the comet assay, IL-1β (10 ng/mL, 24 h) in the IL-8 ELISA assay. *p < 0.05, exposed vs. CTL, n = 3 independent experiments.

None of the PS-COOH particles caused any cell viability loss in both cell systems after 24 h of exposure of non-differentiated cells, while PS-NH_2_ particles triggered a concentration-dependent decrease in cell viability, which was more intense in Caco2-NOD2^*1007fs*^/HT29-MTX (Figure 5A) than in Caco2-NOD2^*WT*^/HT29-MTX (Figure 5B). Indeed, exposure to 50 μg/mL of PS-NH_2_ led to 70% of cell viability loss in Caco2-NOD2^*1007fs*^/HT29-MTX and only 30% in Caco2-NOD2^*WT*^/HT29-MTX (Figure 5A-B). IC50 for PS-NH_2_ was 81.71 ± 8.22 μg/mL in Caco2-NOD2^*WT*^/HT29-MTX (R^2^ = 0.9591) and 18.72 ± 2.16 μg/mL (R^2^ = 0.9798) in Caco2-NOD2^*1007fs*^/HT29-MTX. Neither PS-COOH nor PS-NH_2_ caused any change in intracellular ROS levels (Figure 5C-D), or in the amount of DNA strand breaks (Figure 5E-F). They did not trigger CXCL8 secretion (Figure 5G-H).

Consequently, as expected, non-differentiated cell models are more sensitive towards PS-NH_2_ cytotoxicity than differentiated ones. However, they are more resistant to oxidative stress and DNA damage that could be caused by PS-COOH and PS-NH_2_, compared to differentiated cells.

##### 3.2.2.2 Intracellular accumulation of PS-COOH NPLs

**FIGURE 6:**
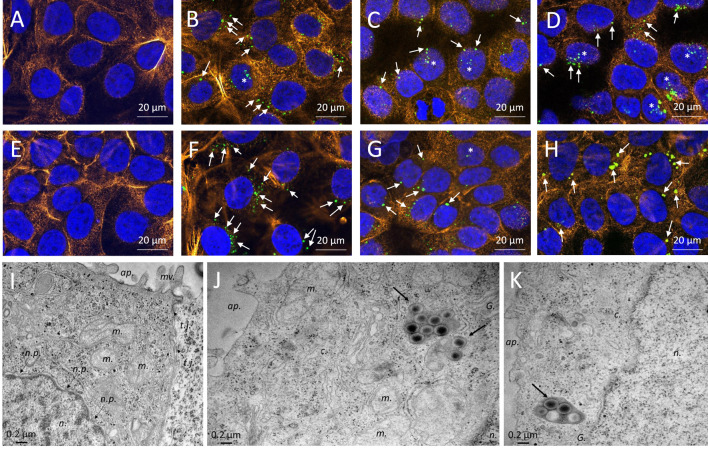
Cellular uptake of PS-COOH particles by non-differentiated Caco2-NOD2/HT29-MTX cells. Confocal **(A–H)** and TEM **(I–K)** images of Caco2-NOD2^*WT*^/HT29-MTX **(A–D,I–K)** or Caco2-NOD2^*1007fs*^/HT29-MTX cells **(E–H)**, either not exposed **(A,E,I)** or exposed to 50 μg/mL of 50 nm **(B,F)**, 100 nm **(C,G)**, 200 nm **(D,H,I,J)** yellow-green fluorescent PS-COOH for 24 h. Blue: DAPI (cell nuclei); orange: phalloïdin-Atto440 (actin network); green: fluorescent PS-COOH particles. Particles are located mainly in the cytoplasm (arrows), rarely in the nucleus (Asterix). Apparent intra-nuclear localization of PS-COOH (Asterisks) is likely attributable to fluorescence bleed-through across z-stacks, as discussed in Supplementary Material (Supplementary Figure S6). Scale = 20 µm. TEM images show cytoplasmic localization of 200 nm PS-COOH particles (arrows), entrapped in electron-dense vesicles. ap.: apical pole, c.: cytoplasm, n.: nucleus, G.: Golgi apparatus, m.: mitochondria, n.p.: nuclear pore, m.v.: microvillosity, t.j.: tight junction. Scale = 200 nm.

In this exposure conditions also, whatever their diameter, fluorescent PS-COOH NPLs accumulated in cells, where they located in the cytoplasm, close to the nucleus, and sometimes inside the nucleus (Figure 6A-H, green dots). Again, in this condition, fluorescent NPLs shined over several z-sections of the sample, suggesting that visualizing them inside the nucleus might be an artefact. TEM confirmed cell uptake of 200 nm PS-COOH particles, but no particle was observed inside cell nuclei (Figure 6I-K). Whatever their size, concentration-dependent accumulation of fluorescent PS-COOH was observed. The amount of accumulated NPLs was similar in both cell models (Supplementary Figure S10). Intracellular accumulation was also confirmed using flow cytometry (Supplementary Figure S11), which also showed concentration-dependent intracellular accumulation, as the percentage of cells having accumulated fluorescence (Supplementary Figure S11A,B) and the mean fluorescence intensity (Supplementary Figure S11C,D) increased with respect to PS-COOH exposure concentration.

##### 3.2.2.3 Impact of PS-COOH particles on the cells’ response to muramyl dipeptide

**FIGURE 7:**
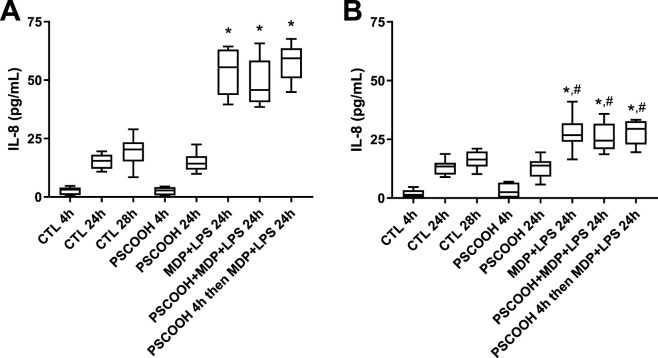
Impact of 50 nm PS-COOH particles on Caco2-NOD2/HT29-MTX cell response to MDP and LPS. Caco2-NOD2^*WT*^/HT29-MTX **(A)** and Caco2-NOD2^*1007fs*^/HT29-MTX **(B)** cells were either not exposed to PS-COOH and grown for 4 h, 24 h, 28h (CTL 4h, CTL 24 h, CTL 28h). Alternatively, they were exposed to PS-COOH for 4 h or 24 h (PS-COOH 4 h and PS-COOH 24 h, respectively) or co-exposed to MPD and LPS or to MDP, LPS and 50 nm PS-COOH for 24 h (MDP+LPS 24 h and PS-COOH+MDP+LPS 24 h, respectively). Finally, they were exposed for 4 h to PS-COOH, then for 24 h to MDP and LPS (PS-COOH 4 h then MDP+LPS 24 h). The release of IL-8/CxCL-8 was monitored via ELISA. Statistical significance, *p < 0.05, exposed vs. respective control, #p < 0.05, Caco2-NOD2^*WT*^/HT29-MTX vs. Caco2-NOD2^*1007fs*^/HT29-MTX, n = 3 independent experiments.

Finally, we assessed the potential of PS-COOH particles to alter the response of NOD2 towards its natural ligand, MDP. This was assessed in Caco2-NOD2^*WT*^/HT29-MTX cells, in order to identify any impairment of NOD2 interaction with MDP and/or any impairment of NOD2 response to MDP. It was also assessed in Caco2-NOD2^*1007fs*^/HT29-MTX cells, which would probe the restoration of interaction between NOD2 and MDP that is constitutively impaired when NOD2 holds the 1007fs mutation. Although it represents only one aspect of NOD2 response to MDP, this was evaluated through the monitoring of CXCL8 (IL-8) secretion through NF-κB activation that is triggered when NOD2 is activated by MDP. Cells were co-exposed to NPLs and a mixture of MDP and LPS either sequentially, *i.e.,* exposed first for 4h to PS-COOH then exposed for 24 h to a mixture of MDP and LPS, or simultaneously, *i.e.,* co-exposed for 24 h to a mixture of PS-COOH, MDP and LPS (Figure 7). Control cells (not exposed) secreted basal levels of CXCL8 after 4 h of growth, which increased 24 h and 28 h post-seeding to reach 10–13 pg/mL CXCL8, in both Caco2-NOD2^*WT*^/HT29-MTX (Figure 7A) and Caco2-NOD2^*1007fs*^/HT29-MTX (Figure 7B). CXCL8 secretion increased in both Caco2-NOD2^*WT*^/HT29-MTX and Caco2-NOD2^*1007fs*^/HT29-MTX when exposed to MDP+LPS, and these increases were both statistically significant. It reached 28 ± 7 pg/mL and 54 ± 10 pg/mL in Caco2-NOD2^*1007fs*^/HT29-MTX and Caco2-NOD2^*WT*^/HT29-MTX, respectively. This increase was significantly higher in Caco2-NOD2^*WT*^/HT29-MTX, compared to Caco2-NOD2^*1007fs*^/HT29-MTX, which was expected as NOD2 is not functional in Caco2-NOD2^*1007fs*^/HT29-MTX due to impaired interaction of NOD2^*1007fs*^ with MDP, thus failing to activate NF-κB.

Exposure for 4 h or 24 h to PS-COOH did not increase or decrease the CXCL8 level, compared to basal CXCL8 level in unexposed cells. Co-exposure to PS-COOH, MDP and LPS for 24 h did not modulate the secretion of CXCL8 as well, compared to cells exposed only to MDP and LPS. Neither did consecutive exposure to PS-COOH for 4h then to MDP and LPS for 24 h change CXCL8 level, compared to cells exposed only to MDP and LPS for 24 h.

As a consequence, we can conclude that in these exposure conditions, PS-COOH showed no impact on NF-κB-mediated CXCL8 secretion, which would be triggered when NOD2 is activated by MDP.

## 4 Discussion

This study evaluated the impact of PS-COOH and PS-NH_2_ NPLs in *in vivo* and *in vitro* models that are representative of healthy population or of individuals with susceptibility to developing Crohn’s disease due to NOD2 polymorphisms. Our focus was on the influence of PS-COOH and PS-NH_2_ NPLs on the pathways through which NOD2 regulates intestinal homeostasis via its interaction with MDP, which leads to inflammation when altered. We focused on endpoints that correspond to known responses of enterocytes and goblet cells in this context, given that the used *in vitro* model was a co-culture of enterocyte-like cells (Caco-2) and goblet-like cells (HT29-MTX).

As previously reviewed (Ferrand et al., 2019), in enterocytes, NOD2 stimulation by MDP triggers the synthesis and release of some antimicrobial peptides (AMPs), whose role is to control the growth and survival of pathogenic bacteria. This response is lost when NOD2 is mutated (Ferrand et al., 2019). NOD2 stimulation is also known to induce the secretion of CXCL8 (IL-8) by enterocytes, thereby triggering the recruitment of neutrophils to the inflammatory site (Ferrand et al., 2019). Moreover, NOD2 activation by MDP normalizes the increase in intestinal paracellular permeability induced by TRL-2- and TRL-4 responses (Ferrand et al., 2019). Goblet cells produce mucus and AMPs that act synergistically to maintain mucus layer homeostasis. Mucus expression is lower in *Nod2* ^*KO*^ mice than in *Nod2* ^*WT*^ mice. There is a reduced number of goblet cells per villus and a reduced number of mucin granules per goblet cell, as well as decreased Muc2 mRNA expression in *Nod2* ^*KO*^ mice compared to *Nod2* ^*WT*^ mice (Ferrand et al., 2019).

Given these established roles of NOD2 in intestinal homeostasis maintenance, we focused the present investigations on monitoring paracellular permeability, mucus homeostasis and the secretion of CXCL-8 and AMP in *in vivo* and *in vitro* models of genetic susceptibility to CD exposed to PS-COOH and PS-NH_2_ NPLs. In addition, in the *in vitro* models, we also evaluated the impact of PS-COOH and PS-NH_2_ NPL on the cells’ oxidative status and on their potential to damage DNA, since these responses have been highlighted in the literature as mechanisms of NPL toxicity.

First, *in vivo*, we observed that single oral exposure to PS-COOH increases intestinal paracellular permeability, as also reported by others (Qiao et al., 2021; Luo et al., 2022; Zheng et al., 2021; Liang et al., 2021; Okamura et al., 2023). Paracellular permeability is regulated by tight junctions, which strictly control the flow of water, ions and small molecules from the luminal compartment to the mucosa when undamaged (Bischoff et al., 2014). It increases when tight junctions are damaged or following cell death via anoïkis, followed by cell shedding, leaving free gaps in the epithelium. Damage to tight junctions may occur if PS-COOH MNPLs reach the intestinal epithelium and accumulate in paracellular spaces. PS-COOH transiting in the intestinal lumen can also act as a mechanical stimulus that stretches epithelial cells, which is known to trigger cell shedding (Bischoff et al., 2014). Paracellular permeability is also increased in inflammatory conditions and in some inflammatory diseases (Johansson et al., 2013), which have been shown by others to be triggered by polystyrene particles (Xu et al., 2021; Luo et al., 2022; Zheng et al., 2021; Xu et al., 2023; Okamura et al., 2023).

We observed this impact only in the ileum of Nod2^*WT*^ mice, while the other parts of the GI tract of Nod2^*WT*^ mice, as well as the whole intestine of Nod2^*KO*^ mice were unaffected. Compared to the colon, the small intestine would be more sensitive to PS-COOH NPLs because it is covered by only one protective mucus layer, whereas the colon has two mucus layers (Johansson et al., 2013). Nevertheless, no impact of PS-COOH was observed in the most proximal parts of the small intestine, i.e., the duodenum and Peyer’s patches, while an impact was observed in the ileum. The mucus layer in the ileum is denser and forms a continuous layer, unlike in the duodenum and jejunum (Ermund et al., 2013), which would rather be in favor of better protection of the ileum. Another difference between the ileum and the other parts of the small intestine is that it is populated by more bacteria. Bacterial dysbiosis is known to cause paracellular permeability perturbation, and PS particles have been described as causing microbiota dysbiosis (Qiao et al., 2021; Liu et al., 2022a; Liu et al., 2022b; Xiao et al., 2022). Therefore, microbiota dysbiosis could also explain why the impact of PS-COOH on paracellular permeability is only observed in the ileum. This perturbation of paracellular permeability is observed only in Nod2^*WT*^ mice. A hypothesis could be that PS-COOH may act on the normal NOD2-mediated attenuating response of enterocytes towards inflammation via normalization of the TRL2/4 responses, which is lacking in Nod2^*KO*^ mice (Barreau et al., 2007). Noteworthy, after exposure to PS-COOH, paracellular permeability in Nod2^*WT*^ mice tends towards that of Nod2^*KO*^ mice.

Our data also show that basal ileal permeability is higher in Nod2KO mice compared to WT mice and that exposure to PS-NH_2_ do not further increase ileal permeability and do not increase villi length. Although the average villus length was not affected by Nod2 deletion (average villus length is similar in Nod2KO and in WT mice), the absence of Nod2 would seem to suppress the impact of PS-NH_2_ on the villi length. Since Nod2 is described to maintain the homeostasis of both intestinal stem cells (Levy et al., 2020; Nigro et al., 2014) and microbiota is described to maintain the integrity of the intestinal epithelium (Mondot et al., 2012), it is possible that in the context of Nod2 deletion, microbiota dysbiosis and altered intestinal stem cell homeostasis could render the epithelium resistant to the impact of PS-NH_2_. In WT mice, as previously reported in chicken, chronic exposure to PS increases the villus area (Mahler et al., 2012). Thus, PS-NH_2_ exposure could exert different effect on the intestinal homeostasis according to the genetic status, then conferring a resistance or an increased susceptibility to develop digestive inflammation such as in Crohn’s disease.

Secondly, we observed decreased mucus content and goblet cell number in mice exposed to PS-COOH and PS-NH_2_, as well as some altered mucin gene mRNA expression under certain conditions. These findings are consistent with those of other authors (Luo et al., 2022; Okamura et al., 2023; Lv et al., 2023). Reduced mucus expression and secretion is one of the manifestations of IBD. Therefore, PS-COOH and PS-NH_2_ particles appear to impart mucus secretion properties to the intestinal mucosa that resemble those of patients with IBD. It has been reported that 40 nm, 100nm and 400 nm PS-COOH particles and 200 nm PS-NH_2_ particles could diffuse through the mucus layer covering HT29-MTX cells, with diffusivity increasing as particle size decreased, and PS-COOH diffusing slightly more than PS-NH_2_ (van Wijngaarden et al., 2025). Larger particles, i.e., 500 nm PS-COOH particles, did not diffuse through this mucus layer, while they were shown by other authors to diffuse through intestinal porcine mucus (Macierzanka et al., 2019). Therefore, it can be hypothesized that PS-COOH particles could reach the ileal epithelium by crossing the weakened mucus layer. We can hypothesize that, then, they would damage tight junctions or cause cell shedding due to mechanical stretching of epithelial cells. Combined with impaired response to inflammatory stimulus, this would aggravate the symptoms of IBD.

Thirdly, we observe impact of PS-COOH on the mRNA expression and secretion of some antimicrobial peptides, which collectively control the microbiota and intestinal colonization by some pathobionts (Bischoff et al., 2014), and which may also affect paracellular permeability (Bischoff et al., 2014). This could also be linked to the microbiota dysbiosis observed in mice exposed to PS particles by others authors (Qiao et al., 2021; Liu et al., 2022a; Liu et al., 2022b; Xiao et al., 2022).

In addition to the observed impact on paracellular permeability, antimicrobial peptides and mucins, we also observed that PS-COOH and PS-NH_2_ particles affected some markers of endoplasmic reticulum stress (ER stress). ER stress has been reported by others following exposure to PS particles (Chen et al., 2024; Yu et al., 2024), and when unresolved, it is known to be central to the IBD condition (Eugene et al., 2020). Nevertheless, this observation should be interpreted with caution as it relies only on the mRNA expression of some factors involved in the unfolded protein response. Therefore, it needs to be confirmed at the protein or at the functional level.

*In vivo* models used in this study are more sensitive to PS-COOH and PS-NH_2_ particles than *in vitro* models. The stronger resistance of *in vitro* intestinal models has already been reported upon exposure to TiO_2_ particles (Brun et al., 2014), and this could be related to the lack of complexity of *in vitro* models, particularly with regard to immune and microbiota components. The mild impact of PS-COOH particles on *in vitro* intestinal models is consistent with current literature (Busch et al., 2021; Domenech et al., 2020; Hesler et al., 2019; Stock et al., 2019). Noteworthy, repeated exposure of Caco-2 cells to PS particles over 8-weeks has been shown to cause changes in cell morphology and to modulate mRNA expression level of oxidative stress-related genes (Domenech et al., 2021). This suggests that longer exposure durations may be necessary to observe toxic impact of PS-COOH and PS-NH_2_ NPLs in such simple *in vitro* models.

No obvious difference in cell response is observed between NOD2^*WT*^ and NOD2^*1007fs*^ *in vitro* model. The only statistically significant responses observed here in the *in vitro* models are i) cytotoxicity of PS-NH_2_ NPLs and ii) mild but significant increase in DNA strand break levels in differentiated Caco2-NOD2^*WT*^/HT29-MTX acutely exposed to 50 μg/mL 50 nm PS-COOH, in both differentiated Caco2-NOD2^*WT*^/HT29-MTX and Caco2-NOD2^*1007fs*^/HT29-MTX acutely exposed to 3.1 μg/mL PS-NH_2_, and in Caco2-NOD2^*1007fs*^/HT29-MTX repeatedly exposed to 50 μg/mL 500 nm PS-COOH. In these conditions, the % DNA in comet tail remains below 2.5%, whereas in control (unexposed) cells, this percentage varies between 0.1 and 0.8. In cells exposed to the positive control, it reaches 50%–80%. While this increase is statistically significant, it is so small that its physiological significance could be considered uncertain.

When NOD2 is mutated to NOD2^*1007fs*^, the protein does not localize to the plasma membrane and remains in the cell cytoplasm, and MDP cannot interact with NOD2 and activate it (Ba et al., 2005). MDP is the natural ligand of NOD2 and their interaction activates the NF-κB pathway (Ferrand et al., 2019). By co-exposing cells to PS-COOH and MDP, we aimed to evaluate whether pre-exposure to PS-COOH would modulate NOD2 response to MDP due to a physiological adaptation, or if PS-COOH particles would bind NOD2 and consequently would impair its binding to MDP, or if MDP would adsorb on PS-COOH, avoiding it proper interaction with NOD2. None of the tested exposure conditions significantly affected CXCL8 secretion, used as a hallmark of NOD2 activation. It suggests that PS-COOH and PS-NH2 NPLs do not affect the interaction between NOD2 and MDP. Nevertheless, impact of PS-COOH NPLs on the other branches of the NOD2 signaling network would need to be tested before a definitive conclusion could be established. Therefore, while the *in vivo* Nod2^*KO*^ model respond to PS-COOH particles in a way that would suggest a link between NOD2 polymorphisms and PS-COOH toxicity, such a link cannot be definitely established based on the responses observed in NOD2-wild-type and NOD2-mutated *in vitro* models.

The fact that these NPLs do not trigger cell death and inflammation in these *in vitro* models would only suggest that they do not impact enterocytes, which are only one cell type involved in the intestinal inflammatory response. Impact of NPLs on immune cells that populate the intestine would have to be tested in order to complement the present result. Moreover, the response of the microbiota to these NPLs would also have to be tested.

## 5 Conclusion

PS-COOH and PS-NH_2_ particles administered orally to mice impact several parameters of intestinal health that are important in the context of IBD, particularly mucus and antimicrobial peptides secretion, paracellular permeability and endoplasmic reticulum stress. The responses of Nod2^*WT*^ and Nod2^*KO*^ mice differ with regard to some, but not all, of the studied endpoints. This suggests that Nod2 status may contribute to the intestinal response to PS-COOH and PS-NH_2_, but does not entirely explain it. *In vitro*, only mild impact of PS-COOH and PS-NH_2_ particles is observed and only in harsh exposure conditions, while PS-COOH accumulates in cells in all tested conditions, suggesting that more complex *in vitro* systems, integrating immune cells and microbiota, could better mimic the intestinal response to PS-COOH observed *in vivo*. Together, these data suggest a minor link between PS-COOH and PS-NH_2_ toxicity and NOD2 polymorphisms. This work would need to be corroborated with other evidences in order to draw clear conclusions on the potential correlation between exposure to nanoplastics and the onset or aggravation of inflammatory conditions in populations with genetic susceptibility to develop Crohn’s disease.

## Data Availability

The datasets presented in this study can be found in online repositories. The names of the repository/repositories and accession number(s) can be found in the article/Supplementary Material.
