# Supplementary material for: Intestinal toxicity of functionalized polystyrene nanoplastics in NOD2 models of Crohn’s disease susceptibility
Source: Front Toxicol. 2026 Jul 21;8:1839932. doi: 10.3389/ftox.2026.1839932 (PMC13432928; doi:10.3389/ftox.2026.1839932)
Supplement: Supplementary file 1 [file DataSheet1.docx]

Supplementary Material for:

Dussol et al., Intestinal toxicity of functionalized polystyrene nanoplastics in NOD2 models of Crohn’s disease susceptibility

# Physico-chemical characterization of PS-COOH and PS-NH_2_ particles

The used particles were supplied as suspensions of PS-COOH with diameters 50, 100, 200, 500 nm, or 50 nm PS-NH_2_. Their primary diameter was estimated by analysis of transmission electron microscopy (TEM) images, recorded using negative stain on grid. Briefly, samples were deposited to the clean side of a carbon film on mica, stained and transferred to a 400-mesh copper grid. Images were recorded using a Tecnai 12 LaB6 EM operating at 120 kV, equipped with a Gatan Orius 1000 CCD Camera. Their size distribution and zeta potential were measured via dynamic light scattering (DLS) and electrophoretic light scattering (ELS) using a nanoZS zetasizer (Malvern). Moreover, absence of endotoxin contamination was proven using Pierce chromogenic endotoxin contamination assay (thermoFisher Scientific).

The results are the following. TEM images, reported in Figure S1A-E, show that MNPLs appeared to be slightly smaller than expected, i.e., 37 nm, 91 nm, 145 nm, 402 nm (PS-COOH) and 36 nm (PS-NH_2_), respectively, instead of 50 nm, 100 nm, 200 nm, 500 nm (PS-COOH) and 50 nm (PS-NH_2_) (Table S2). As expected, all these particles formed stable suspension in water at 100 µg/mL, with Z-averages of 52±1 (PdI 0.049±0.004) and 59±1 nm (PdI 0.094±0.019) for 50 nm PS-COOH and 50 nm PS-NH2, respectively. The Z-averages were 52±1 (PdI 0.049±0.004), 108±1 (PdI 0.040±0.029), 211±2 (PdI 0.026±0.006) and 551±6 (PdI 0.083±0.012), for PS-COOH 50 nm, 100 nm, 200 nm and 500 nm, respectively, and 59±1 (PdI 0.094±0.019) for PS-NH_2_ (Table S2).

PS-COOH and PS-NH_2_ particle size distributions in water and in cell culture medium are reported in Figure S1F-G. As reported in Table S2, the zeta potential of PS-COOH was slightly negative, while that of PS-NH_2_ particles was close to zero but positive. These suspensions showed a very good colloidal stability although their zeta potential was between -15 mV and 15 mV, which is generally considered as a range of zeta potential where particles are not stable {Hiemenz, 1997 #170}. This suggests that these MNPLs are stabilized with a surfactant, possibly a nonionic surfactant such as Triton-X100, which provides steric repulsion, counterbalancing the absence of electrostatic repulsion. Upon dilution in cell culture medium, size distributions and Z-averages shifted to higher values, showing that particles either slightly agglomerated and/or got coated with a biomolecular corona. Their PdI remained close to 0.1 except for 50 nm PS-COOH and PS-NH_2_ particles, which showed PdI of 0.646 and 0.511, respectively. These results suggest that the slight increase of Z-average observed with 100 nm, 200 nm and 500 nm was probably rather due to surface corona formation as low PdI values indicate stable suspensions. As for 50 nm particles, the PdI increase could originate from both biocorona formation and from particle agglomeration. None of the used particles was tested positive for endotoxin contamination (Figure S1H).

**Supplementary Table 1.** Z-average and zeta potential of the PS particles*^a^*

|  | PS-COOH  50 nm | PS-COOH  100 nm | PS-COOH  200 nm | PS-COOH  500 nm | PS-NH2  50 nm |
| --- | --- | --- | --- | --- | --- |
| Prim. diameter | 37±10 | 91±7 | 145±17 | 402±14 | 36±6 |
| Z-ave., water | 52 ± 1 | 108 ± 1 | 211 ± 2 | 551 ± 6 | 59 ± 1 |
| PdI, water | 0.049 ± 0.004 | 0.040 ± 0.029 | 0.026 ± 0.006 | 0.083 ± 0.012 | 0.094 ± 0.019 |
| Z-ave, medium | 171 ± 7 | 140 ± 1 | 308 ± 6 | 560 ± 4 | 171 ± 2 |
| PdI, medium | 0.646 ± 0.047 | 0.105 ± 0.006 | 0.194 ± 0.015 | 0.185 ± 0.016 | 0.511 ± 0.009 |
| ζ (mV) | -32.1 ± 0.7 | -37.2 ± 0.7 | -46.3 ± 0.3 | -48.4 ± 0.4 | 3.3 ± 0.1 |

*^a^*Z-average and zeta potential were measured using a Malvern NanoZS zetasizer. The primary diameter (Prim.diameter) and Z-averages (Z-ave) are expressed in nm. The Z-average and polydispersity indexes (PdI) values were derived from three analyses of a 100 µg/mL particle suspension, either diluted in water (Z-average, water and PdI, water) or in cell culture medium (Z-average, medium and PdI, medium). The zeta potential (ζ) was measured in ultrapure water to which was added a drop of 150 mM NaCl to ensure conductivity.


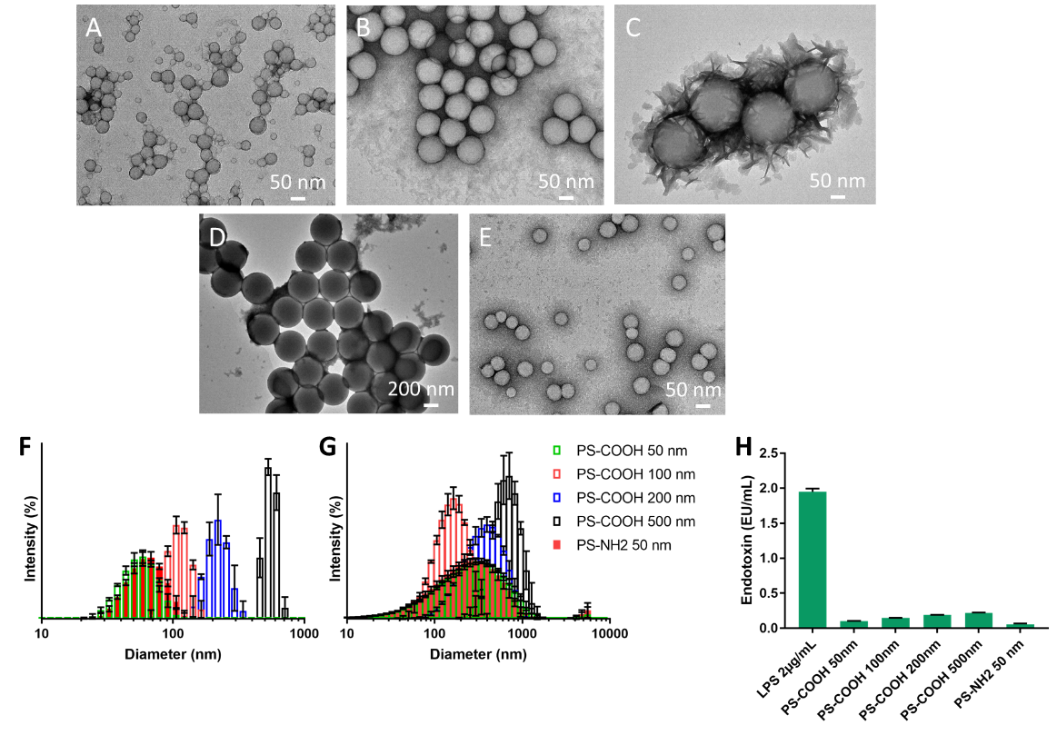


**Supplementary Figure 1**. Shape and size of PS particles. TEM images of 50 nm (A), 100 nm (B), 200 nm (C) and 500 nm (D) PS-COOH particles, and of 50 nm PS-NH_2_ particles (E). Size distribution measured via dynamic light scattering on PS particles diluted either in water (F) or in complete cell culture medium (G). Values are derived from Intensity measurements, on 100 µg/mL particle suspensions. Endotoxin contamination, determined using the Limulus amoebocyte lysate (LAL) assay (H). Mean ± standard deviation, n=3 measurements.

# Characterization of the Caco2-NOD2*^WT^* and Caco2-NOD2*^1007fs^* cell lines

*In vitro* models of genetic susceptibility to CD were constructed, by transduction of Caco-2 cells with a lentivirus encoding the 1007 frameshift mutation (1007fs) of NOD2 gene (NOD2*^1007fs^*), giving rise to a cell line stably expressing NOD2*^WT^*. To avoid any misinterpretation of any effect that would derive from the transduction itself, a control cell line was constructed in which Caco-2 cells were transduced with wild-type NOD2 (NOD2*^WT^*).

First, the transduction efficiency of plasmids carrying NOD2*^WT^* or NOD2*^1007fs^* gene was validated by measuring NOD2 mRNA expression in both cell lines. As NOD2*^WT^* or NOD2*^1007fs^* differ only by a frameshift mutation, they cannot be distinguished by mRNA expression analysis, *i.e*., the mRNA expression of NOD2 reflects that of both gene variants. NOD2 mRNA expression was increased in both transduced cell lines, compared to commercial Caco-2 cells, with an absolute gene regulation factor of 9.8±3.4 and 2.8±1.2 in Caco2-NOD2*^WT^* and Caco2-NOD2*^1007fs^*, respectively (Figure S2A). This confirms that both NOD2*^WT^* or NOD2*^1007fs^* genes had been efficiently incorporated in actively transcribed sites of the genome. NOD2*^WT^* mRNA expression was stronger in Caco2-NOD2*^WT^* compared to NOD2*^1007fs^* in Caco2-NOD2*^1007fs^*, probably due to a more efficient incorporation of NOD2*^1007fs^* in the genome of Caco2 cells. As expected [1], NOD2 expression was also significant in non-transduced Caco2 cells, but it was much lower. NOD2 expression in transduced cells was stable over time as these RT-qPCR experiments were performed at passage 20 and 23 after the transduction of Caco2-NOD2*^WT^* and Caco2-NOD2*^1007fs^* cells, respectively.

Then, the functionality of transduced genes was validated by evaluating the activation of NF-κB pathway upon stimulation with MDP and LPS, MDP being the natural ligand of NOD2 that triggers activation of NF-κB, and LPS being known to enhance NOD2 expression [1]. As expected, exposure for 24 h to 1 or 5 µg/mL MDP significantly increased CXCL8 secretion in Caco2-NOD2*^WT^* cells (Figure S2B, blue bars), proving activation of the NF-κB pathway, while it did not increase in Caco2-NOD2*^1007fs^* cells (Figure S2C, blue bars). Co-exposure to MDP and LPS further increased CXCL8 secretion by Caco2-NOD2*^WT^* compared to exposure to MDP only (Figure S2B, green bars). Co-exposure to MDP and LPS triggered a slight and non-significant response in Caco2-NOD2*^1007fs^* as well (Figure S2C, green bars), which can be attributed to LPS since the same level of CXCL8 secretion was observed in Caco2-NOD2*^WT^* cells exposed to LPS only (Figure S2B, yellow bar).


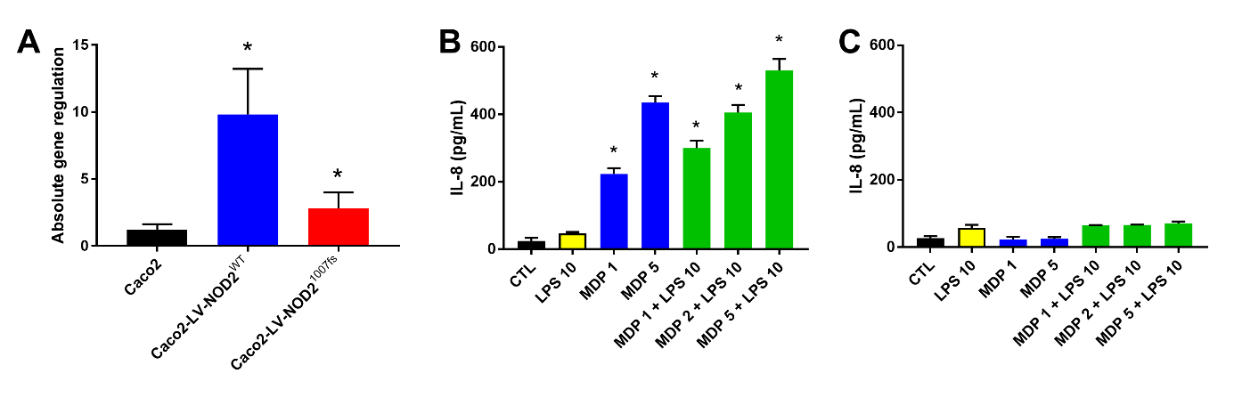


**Supplementary Figure 2**. Validation of the Caco2-NOD2*^WT^* and Caco2-NOD2*^1007fs^* cell lines. mRNA expression was measured in Caco2, Caco2-NOD2*^WT^* and Caco2-NOD2*^1007fs^* cell lines (A). IL-8/CxCL-8 secretion was measured in Caco2-NOD2*^WT^* (B) and Caco2-NOD2*^1007fs^* (C) exposed to MDP at 1 µg/mL (MDP 1 and MDP 1 + LPS 10), 2 µg/mL (MDP 2 + LPS 10) or 5 µg/mL (MDP 5 and MDP 5 + LPS 10) and LPS at 10 µg/mL (MDP 1 + LPS 10, MDP 2 + LPS 10 and MDP 5 + LPS 10). Mean ± standard deviation, n=3 measurements. Statistical significance, *p<0.05 exposed vs. control (unexposed cells).

# Additional statistical analysis of *in vivo* results

Figure 1 suggests that Nod2*^KO^* mice may have a higher intestinal paracellular permeability than WT mice. This was verified and results are reported in Supplementary Figure 3.

**Supplementary Figure 3**. Comparison of the intestinal paracellular permeability in WT and Nod2*^KO^* mice. Paracellular permeability was measured in the duodenum, Peyer’s patches, ileum and colon of WT and Nod2*^KO^* mice. Results are expressed as mean +/- sem. Two-group comparisons were performed using unpaired t-tests, assuming that data followed a Gaussian distribution, which was verified using a Kolmogorov-Smirnov test. Statistical significance: *P<0.05, **P<0.01, ****P<0.001. All P values indicated are 2-tailed.

# Messenger RNA (mRNA) expression

Messenger RNA expression was assessed using RT-qPCR. The primer sequences reported in Table S2 were used in the reaction.

**Supplementary Table 2.** RT-qPCR primer sequences

| Gene | Forward | Reverse |
| --- | --- | --- |
| *Human* |  |  |
| NOD2 | ATGGGCTTTGATGGGGGAAG | AGCACATTTCACAACCCGGA |
| *Mouse* |  |  |
| Gapdh | AGGTCGGTGTGAACGGATTTG | TGTAGACCATGTAGTTGAGGTCA |
| Act | CCTGGCACCCAGCACAAT | GCCGATCCACACGGAGTACT |
| Il1-β | ACCTTCCAGGATGAGGACATGAG | CATCCCATGAGTCACAGAGGATG |
| Tnf-α | CCACGCTCTTCTGTCTACTGCAC | GGTCTGGGCCATAGAACTGAT |
| Ifn-γ[2] | CAGCAACAGCAAGGCGAAA | AGCTCATTGAATGCTTGGCG |
| Il-12[2] | ACGAGAGTTGCCTGGCTACTAG | CCTCATAGATGCTACCAAGGCAC |
| Zo1 | CAGCCTCCAGAGTTTGACAG | TCCACAGCTGAAGGACTCACAG |
| Ocln | GAGTGAAGAGTACATGGCTGCT | TTCTCCCGCAACTGGCATC |
| Cldn2 | CCCACAGATACTTGTAAGGAG | CCAAAAGGCCTAGGATGTAG |
| Muc2 | TGACTGCCGAGACTCCTACA | CTGTAGTGTGGGGTGCTGAC |
| Muc4 | TGGCTACAAAGGCTACCACC | CCCTCACTACATGGGGACAC |
| Reg3γ | TCTCAGGTGCAAGGTGAAGTT | AATAGGAGCCATAGGCACGG |
| Reg3β | GGTTTGATGCAGAACTGGCCT | TGAGCACAGATACGAGGTGTCC |
| Tff3 | ACGTTGGCCTGTCTCCAAG | GGAGCCTGGACAGCTTCAAA |
| Lys1p[3] | GCCCAGGCCAAGGTCTACAAT | ATGCTCGAATGCCTTGGGGA |
| Mmp7 | TTTGATGGGCCAGGGAACACTCTA | ATGGGTGGCAGCAAACAGGAAGT |
| Mlck | AATTCATCGTCCTCGCCCAA | CTCACTGGTCTGAGCATCGTC |
| Cntrl | GAGGAATCGTTGAGAGATCTGGTATG | CCATGTTCAGCGACAGCAGAGC |
| Allcrypt | ATGAAGAGACTTGTCCTCCTC | CTTCTTGAAGAGCAGAGCCTT |
| Grp94[4] | AAGAATGAAGGAAAAACAGGACAAAA | CAAATGGAGAAGATTCCGCC |
| Bip[4] | TTCAGCCAATTATCAGCAAACTCT | TTTTCTGATGTATCCTCTTCACCAGT |
| totXbp1[4] | CAGCACTCAGACTATGTGCA | GTCCATGGGAAGATGTTCTGG |
| usXbp1[4] | CAGCACTCAGACTATGTGCA | GTCCATGGGAAGATGTTCTGG |
| Chop[4] | CCACCACACCTGAAAGCAGAA | AGGTGAAAGGCAGGGACTCA |
| Atf4[5] | GCAAGGAGGATGCCTTTTC | GTTTCCAGGTCATCCATTCG |
| Atf6[5] | GGACGAGGTGGTGTCAGAG | GACAGCTCTTCGCTTTGGAC |

**Supplementary Table 3.** mRNA expression analysis in the ileum of mice exposed to PS-COOH and PS-NH_2_ particles*^a^*

|  |  | WT |  | WT |  | Nod2^KO^ |  | Nod2^KO^ |  |
| --- | --- | --- | --- | --- | --- | --- | --- | --- | --- |
|  |  | PSCOOH |  | PSNH_2_ |  | PSCOOH |  | PSNH_2_ |  |
|  | Gene | Expr. | *95% C.I.* | Expr. | 95% C.I. | Expr. | *95% C.I.* | Expr. | *95% C.I.* |
| *Inflammation* | *Ifnγ* | 0,636 | *0,196 - 1,343* | - | - | 0,502 | *0,258 - 0,976* | - | - |
|  | *Il-1β* | 0,584 | *0,085 - 1,837* | - | - | - | - | - | - |
|  | *Tnfα* | - | - | - | - | 3,422 | *1,511 - 8,618* | 0,594 | *0,070 - 3,267* |
|  | *Il-12* | - | - | - | - | 5,995 | *2,247 - 24,193* | - | - |
| *Mucus* | *Muc2* | - | - | - | - | 0,489 | *0,22 - 1,142* | 0,446 | *0,004 - 1,951* |
|  | *Muc4* | 0,678 | *0,232 - 1,686* | - | - | 0,589 | *0,346 - 1,013* | - | - |
|  | *Tff3* | 0,441 | *0,078 - 1,558* | 0,747 | *0,224 - 1,873* | - |  | 0,628 | *0,161 - 1,409* |
| *Antimicrobial peptides* | *Mmp7* | 0,447 | *0,068 - 1,817* | *-* | *-* | 0,244 | *0,069 - 0,565* | 0,597 | *0,136 - 1,460* |
|  | *Reg3β* | 0,259 | *0,034 - 1,654* | 0,661 | *0,194 - 1,963* | 0,391 | *0,096 - 1,08* | 0,441 | *0,011 - 6,431* |
|  | *Reg3γ* | 0,241 | *0,044 - 0,708* | - | *-* | 0,382 | *0,053 - 2,754* | - | - |
|  | *Lyz1p* | - | - | - | *-* | 0,382 | *0,14 - 1,314* | 0,53 | *0,063 - 3,173* |
|  | *AllCrypt* | - | - | - | *-* | 0,052 | *0,011 - 0,276* | - | - |
| *UPR response* | *Grp94* | 0,471 | *0,14 - 1,211* | 0,779 | *0,312 - 1,571* | 0,76 | *0,452 - 1,095* | - | - |
|  | *Bip* | 0,586 | *0,185 - 1,707* | 0,59 | *0,254 - 1,426* | - | - | 0,563 | *0,040 - 1,989* |
|  | *Atf4* | 0,63 | *0,289 - 1,177* | *-* | *-* | 0,541 | *0,284 - 1,328* | 0,644 | *0,065 - 3,222* |
|  | *Chop* | - | - | - | *-* | 1,481 | *0,966 - 2,673* | - | - |
|  | *Atf6* | - | - | - | *-* | 0,595 | *0,273 - 1,345* | - | - |
|  | *tot Xbp1* | 0,495 | *0,171 - 1,145* | 0,855 | *0,525 - 1,364* | 0,465 | *0,212 - 1,264* | - | - |
|  | *us Xb1* | 0,49 | *0,161 - 1,153* | - | -- | 0,564 | *0,226 - 1,681* | - | - |
| *Cell junctions/intestinal* | *Cldn2* | - | - | - | - | 0,691 | *0,339 - 1,139* | - | - |
| *permeability* | *Ocln* | - | - | - | - | - | - | 1,312 | *0,510 - 2,874* |
|  | *Zo1* | - | - | - | - | - | - | 1,431 | *0,632 - 2,940* |
|  | *Mlck* | 0,65 | *0,346 - 1,305* | - | *-* | - | - | 0,448 | *0,002 - 2,891* |
|  |  |  |  |  |  |  |  |  |  |

*^a^*This table shows absolute gene regulation (Expr.) and 95% confidence interval (95% CI) calculated with the ΔΔCt method, using REST2009. Non-statistically significant results are marked with a dash, whereas for statistically significant results, the change in mRNA expression is indicated, together with the 95% confidence interval (in italics).

# Additional *in vitro* data

Transepithelial resistance (TEER) was measured after 24 h of exposure of differentiated Caco2-NOD2*^WT^*/HT29-MTX and differentiated Caco2-NOD2*^1007fs^*/HT29-MTX to PS-COOH (50 and 500 nm) or to PS-NH_2_ (50 nm). Only the positive control (EDTA) triggered a statistically significant decrease of the TEER, while the particles did not significantly change the TEER (Supplementary Figure 4).


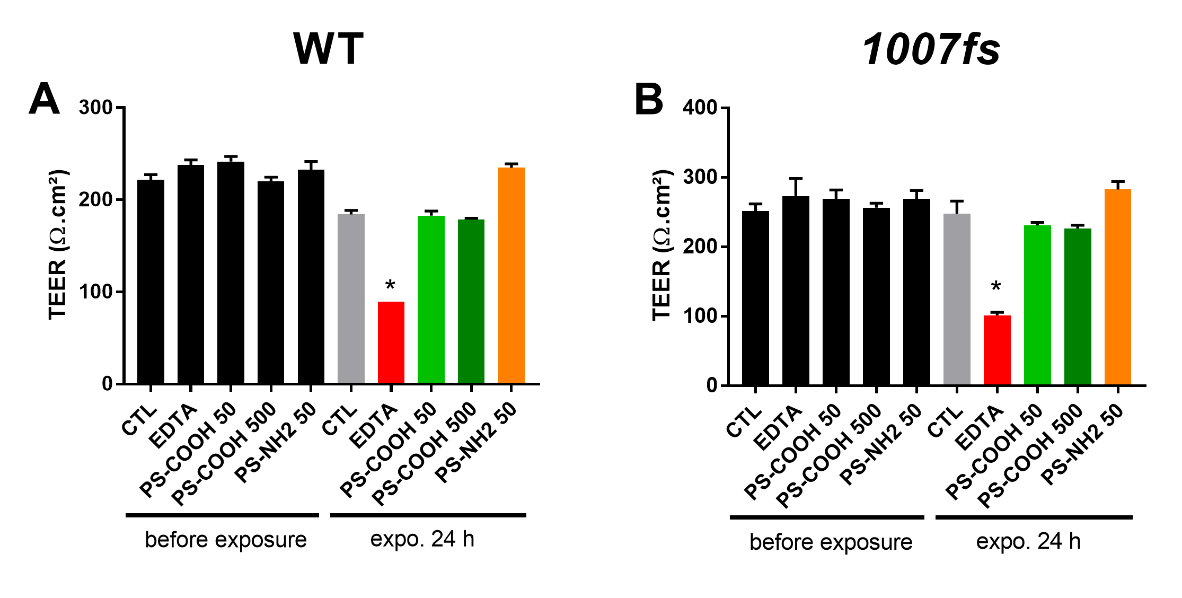


**Supplementary Figure 4**. Transepithelial resistance (TEER). TEER was measured in differentiated Caco2-NOD2*^WT^*/HT29-MTX and Caco2-NOD2*^1007fs^*/HT29-MTX, before exposure to any particle (“before exposure”) or after 24 h of exposure to 50 nm or 500 nm PS-COOH or to 50 nm PS-NH_2_ (expo. 24 h). Statistical significance: *p<0.05, exposed vs. CTL, n=3.

Intracellular accumulation of fluorescent PS-COOH particles was probed by confocal microscopy and by fluorescence measurement using a microplate reader or using flow cytometry.


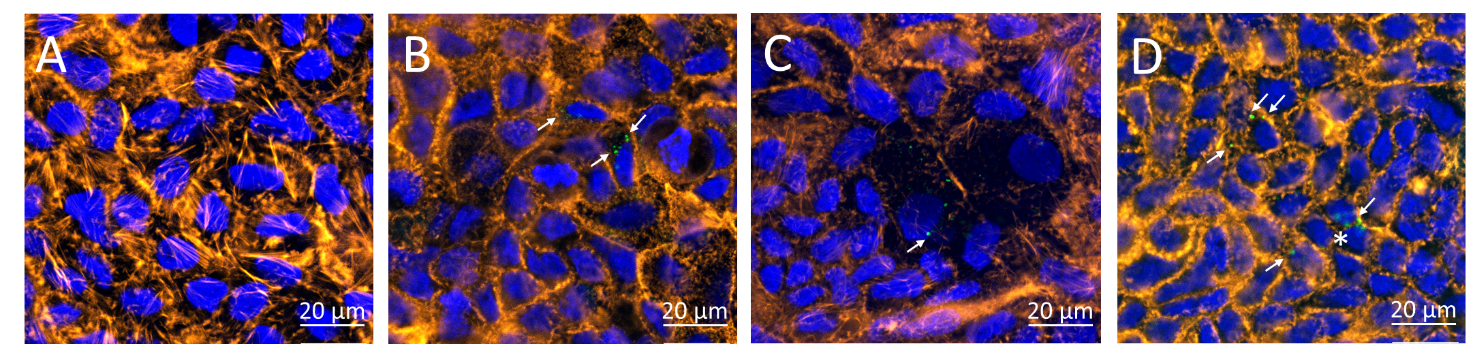


**Supplementary Figure 5**. Intracellular accumulation of PS-COOH particles in differentiated Caco2-NOD2*^1007fs^*/HT29-MTX cells, after 24 h of exposure. Confocal images of Caco2-NOD2*^1007fs^*/HT29-MTX cells either not exposed (A) or exposed to 50 µg/mL of 50 nm (B), 100 nm (C) or 200 nm (D) yellow-green fluorescent PS-COOH for 24 h. Blue: DAPI (cell nuclei); orange: phalloïdin-Atto440 (actin network); green: fluorescent PS-COOH particles. Particles are located mainly in the cytoplasm (arrows), rarely in the nucleus (asterix).


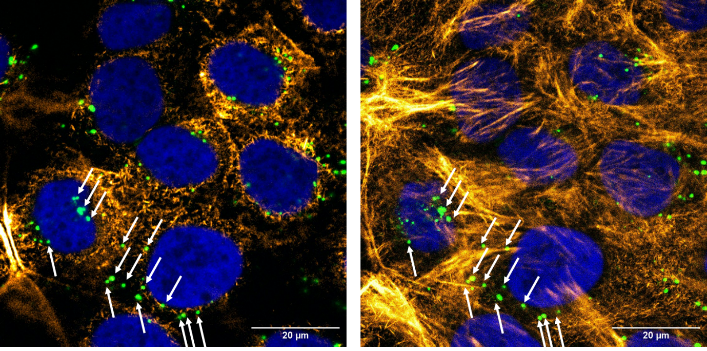


**Supplementary Figure 6**. Confocal images of Caco-2/HT29-MTX cells having accumulated fluorescently-labelled PS-COOH particles. Agglomerated of PS-COOH particles could be observed on several successive confocal stacks, suggesting that their fluorescence was so brilliant that it could shine even on stacks that did not correspond to locations of PS-COOH particles.

**Supplementary Figure 7**. Accumulation of fluorescent PS-COOH particles in differentiated Caco2-NOD2*^WT^* or Caco2-NOD2*^1007fs^* cells cocultured with HT29-MTX, after acute exposure. Caco2-NOD2*^WT^* /HT29-MTX (A) and Caco2-NOD2*^1007fs^*/HT29-MTX (B) cells were exposed to 12.5, 25 or 50 µg/mL green fluorescent 50 nm, 100 nm, 200 nm or 500 nm PS-COOH, for 24 h after post-confluence differentiation.

**Supplementary Figure 8**. Accumulation of fluorescent PS-COOH particles in differentiated Caco2-NOD2*^WT^* or Caco2-NOD2*^1007fs^* cells cocultured with HT29-MTX, after repeated exposure. Caco2-NOD2*^WT^* /HT29-MTX (A) and Caco2-NOD2*^1007fs^*/HT29-MTX (B) cells were exposed to a total of 12.5, 25 or 50 µg/mL green fluorescent 50 nm, 100 nm, 200 nm or 500 nm PS-COOH, repeatedly for 4 successive days (1/4^th^ the dose each day) after post-confluence differentiation.


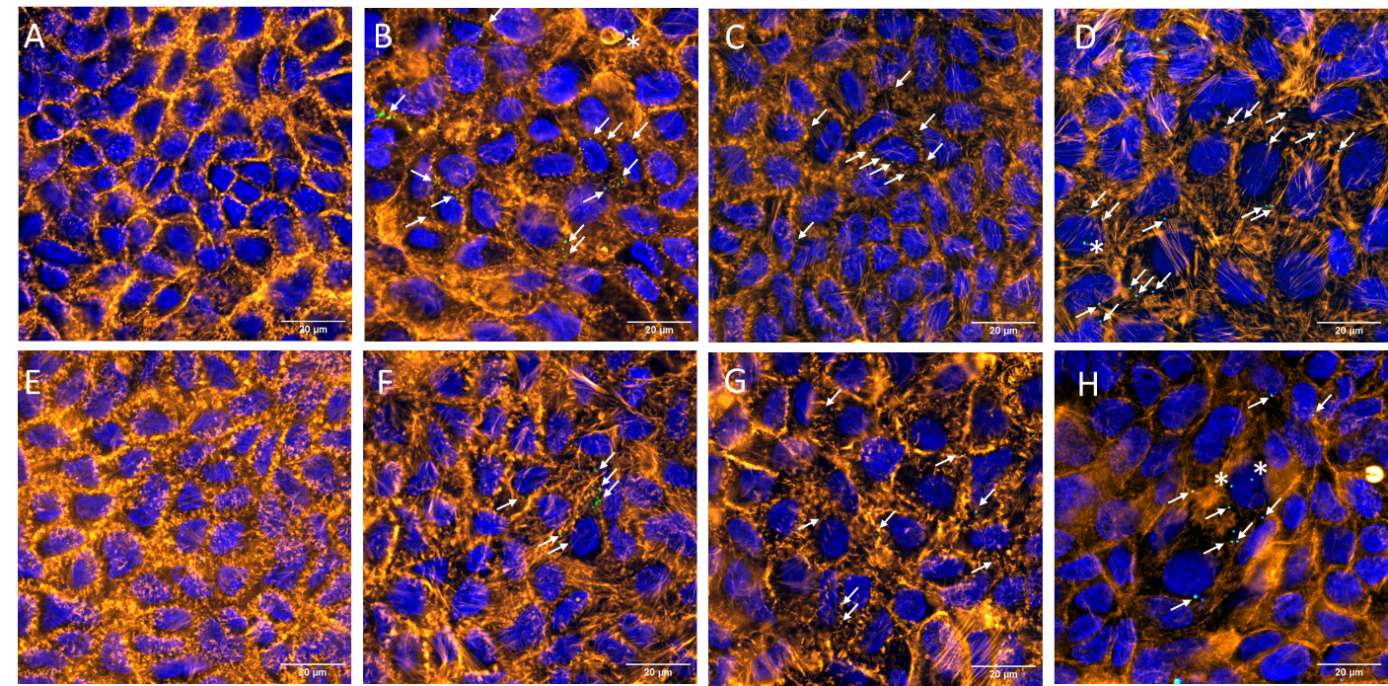


**Supplementary Figure 9**. Intracellular accumulation of PS-COOH particles in differentiated Caco2-NOD2/HT29-MTX cells, upon repeated exposure. Confocal images of Caco2-NOD2*^WT^*/HT29-MTX (A-D) or Caco2-NOD2/HT29-MTX cells (E-H), either not exposed (A, E) or exposed to 50 µg/mL of 50 nm (B, F), 100 nm (C, G) or 200 nm (D, H) yellow-green fluorescent PS-COOH for 24 h. Blue: DAPI (cell nuclei); orange: phalloïdin-Atto440 (actin network); green: fluorescent PS-COOH particles. Particles are located mainly in the cytoplasm (arrows), rarely in the nucleus (stars).

**Supplementary Figure 10**. Accumulation of fluorescent PS-COOH particles in non-differentiated Caco2-NOD2*^WT^* or Caco2-NOD2*^1007fs^* cells cocultured with HT29-MTX, after acute exposure. Caco2-NOD2*^WT^* /HT29-MTX (A) and Caco2-NOD2*^1007fs^*/HT29-MTX (B) cells were exposed to a total of 12.5, 25 or 50 µg/mL green fluorescent 50 nm, 100 nm, 200 nm or 500 nm PS-COOH, for 24 h one day after seeding (non-differentiated).

**Supplementary Figure 11**. Flow cytometry analysis of PS-COOH accumulation in Caco-2/HT29-MTX cells. Accumulation of 50 nm, 200 nm and 500 nm yellow-green fluorescent PS-COOH particles was assessed in Caco2-NOD2*^WT^*/HT29-MTX or Caco2-NOD2*^1007fs^*/HT29-MTX cells, exposed to 12.5, 25 or 50 µg/mL of the particles, via flow cytometry. After 24 h of exposure, the % of cells showing yellow-green fluorescence (Figure S4A-B), representative of cells having accumulated yellow-green fluorescent particles, and the mean fluorescence intensity (MFI) (Figure S4C-D) was measured in Caco2-NOD2*^WT^*/HT29-MTX (Figure S4A, C) or Caco2-NOD2*^1007fs^*/HT29-MTX (Figure S4B, D). Statistical significance, *p<0.05 exposed vs. control (unexposed cells).

# References

[1] Thiébaut, R., et al., *Characterization and Genetic Analyses of New Genes Coding for NOD2 Interacting Proteins.* PLoS One, 2016. **11**(11): p. e0165420.
